# Technology-Facilitated Gender-Based Violence Against Politically Active Women: A Systematic Review of Psychological and Political Consequences and Women’s Coping Behaviors

**DOI:** 10.1177/15248380251343185

**Published:** 2025-06-27

**Authors:** Luise Koch, Maria Paula Russo Riva, Janina Isabel Steinert

**Affiliations:** 1 Technical University of Munich, Germany; 2 Secretariat of Social Development of the State of São Paulo, Brazil; 3 Munich Center for Health Economics and Policy, Germany

**Keywords:** technology- or image-based abuse, gender-based violence, health effects, internet and abuse, mental health effects

## Abstract

Technology-facilitated gender-based violence presents critical challenges for politically active women, whose professional roles often expose them to elevated levels of online abuse with far-reaching impacts on their emotional well-being, professional engagement, and participation in public life. This systematic review synthesizes findings from 48 studies employing qualitative, quantitative, and mixed-methods research to examine the psychological and political consequences and coping mechanisms associated with online harassment. Eighty-one percent of the included studies (39/48) report psychological distress, anxiety, and fear among targeted women, with 31% of the studies (15/48) identifying online harassment as a trigger for (re-) traumatization. The political consequences are equally significant, with 62% of the studies (30/48) documenting modifications in political messaging, 39% (19/48) noting reduced engagement with online platforms, and 29% (14/48) showing that women abandon their online presence altogether. Additionally, 20% (10/48) of the studies report cases of women withdrawing from their political roles. In terms of coping strategies, 66% (32/48) report women blocking or muting harassers, while 37% (18/48) document women reporting abuse to authorities or platforms. This study highlights the pervasive impact of technology-facilitated violence on women’s emotional well-being, and their political participation and underscores its broader implications for democratic discourse and social equity.

## Introduction

Technology-facilitated gender-based violence (TFGBV) has emerged as a significant concern in the digital age, with the potential to cause substantial emotional, professional, and political harm to women (E. A. Jane, 2018; Krook & Sanín, 2020; Southern & Harmer, 2021; Ward & McLoughlin, 2020). Women who are publicly visible and challenge existing societal structures—that is to say, politicians, activists, and journalists reporting on political or activist topics—face attacks that aim to silence them (Löfgren Nilsson & Örnebring, 2016). Across different regional and methodological contexts, the prevalence of online abuse toward politically active women is consistently high. Survey data show that 50.2% of politically active women in Colombia, 22.7% in Kenya, and 17.6% in Indonesia report having experienced online violence (Zeiter et al., 2019). In the United Kingdom, 62% of Members of Parliament (MPs) reported receiving at least one abusive tweet over a 2-month period (Ward & McLoughlin, 2020), while another study found that 91.8% of MPs experienced online harassment weekly or daily (Akhtar & Morrison, 2019). Complementing these quantitative patterns, qualitative research also reveals near-daily harassment of female journalists from news audiences on platforms like X and Facebook (Holton et al., 2021). These findings confirm that online abuse is not an isolated experience but a structural feature of digital political spaces for women in public life.

The consequences of such attacks can be far-reaching, affecting not only the visibility and professional advancement of women in public spheres but also their mental health and sense of safety (Krook & Sanín, 2020; Posetti & Shabbir, 2022). In this context, TFGBV has the potential to impede women’s ability to engage fully with digital platforms, to network or market themselves, as well as to participate in the self-expression and representation that were previously regarded as fundamental benefits of the Web 2.0 era. This, in turn, limits women’s participation in political spaces and discourages others from entering or renewing their involvement in these arenas. Prior research indicates that being exposed to online abuse can have a deterrent effect on women’s political ambitions and their participation in public life (Koch et al., 2024; Wagner, 2022). Furthermore, it has a spillover effect, discouraging other women from pursuing political office (Haraldsson & Wängnerud, 2019; Southern, 2024; Vrielink & van der Pas, 2024).

Despite a substantial body of work examining the prevalence and forms of TFGBV, fewer studies have employed a systematic approach to examining the range of emotional and political consequences, as well as the potential coping mechanisms employed by women facing it. In this review, TFGBV is defined as the deliberate use of technological tools—including but not limited to social media, messaging apps, or surveillance technologies—to harm, threaten, coerce, or silence women in public-facing roles (Koukopoulos et al., 2025; UNESCO, 2023). This includes manifestations such as trolling, impersonation, targeted threats, stalking, intimate sexual abuse, or doxing. Although the conceptualization of “technology” within this field is broad, the majority of the existing research has focused primarily on social media platforms due to their prominence in public and political discourse. This review reflects that emphasis, while remaining grounded in a definition of TFGBV that extends across the broader digital ecosystem. It can be reasonably assumed that politically active women may be a particularly vulnerable group, given their visibility and the fact that their public and online presence is a key requirement of their job. Against this background, this paper aims to synthesize existing evidence from both qualitative and quantitative studies in order to gain insight into how politically active women respond to digital harassment. In particular, this review aims to focus on (a) the psychological or emotional consequences, (b) the political or professional consequences of digital harassment for their professional practices, and (c) the coping mechanisms that they employ.

The psychological and political consequences of harassment, as well as the applied coping mechanisms, cannot simply be treated as a general whole. Previous studies on the psychological consequences of online hate reveal predominantly negative reactions on the part of women targeted, with relatively few reports of defiant reactions. In a survey of 181 UK politicians conducted by Akhtar and Morrison (2019), it was revealed that online hate had a significant psychological impact on female politicians, with high levels of worry and concern about their safety. Similarly, Ferrier’s (2018) research showed how online harassment can serve as a trigger for re-traumatization, especially for women with previous experiences of violence. In the same study, respondents reported strong physical reactions—such as heart pounding, trouble breathing, and sweating—when reminded of the stressful experience, emphasizing the severe emotional toll. Conversely, Lewis et al. (2016, p. 14), discovered that 54% (*n* = 226) of activists reported feeling more resolute in their political views after experiencing online hate, with 34% indicating that they felt motivated to take action in response to the attacks they had received.

In examining the political consequences of online harassment for targets of TFGBV, prior research has identified that they are severe and far-reaching, affecting both the final products of women’s work and the routines required to produce them (Holton et al., 2021). Women may employ a range of protective behaviors, including deleting social media profiles, or avoiding voicing their opinion in potentially hostile environments (Binns, 2017; Tandoc et al., 2023). The various forms of self-censorship not only constrain individual participation in political discourse but also curtail the diversity of voices engaged in it, which can have long-term implications for democratic engagement. The additional burden of this preventive labor, including the decision-making processes involved in determining which platforms are safer and whether some specific content is suitable for posting, introduces another layer of complexity to women’s professional lives (Sobieraj, 2021). These additional responsibilities necessitate balancing dual obligations pertaining to public engagement and self-protection.

Previous studies have found that politically active women employ a range of coping strategies to manage the emotional and professional impact of TFGBV. In response to online harassment, individuals may engage in either “flight” or “fight” coping mechanisms, as proposed by E. A. Jane (2016). “Flight” responses in digital contexts often involve various forms of withdrawal or disengagement, such as muting or blocking abusers, reducing participation in online discussions, avoiding certain topics, or even deactivating social media accounts (Carlson & Witt, 2020; Eckert, 2017; Jane, 2017; Lewis et al., 2016). Similarly, the strategy of “purposeful ignoring” is a common tactic employed by some women who avoid comments or feedback altogether in order to maintain their psychological well-being (Sobieraj, 2021). In contrast, “fight” responses entail confronting the perpetrators of abuse, whether online or offline. This may entail publicly calling them out or exposing them to their followers or a wider audience as a form of resistance (E. A. Jane, 2016). Reporting to platforms or authorities can be understood as another form of “fight” response, especially when framed as an attempt to demand accountability or institutional consequences (Jane, 2017). These coping mechanisms are essential for individual survival in hostile online environments, but also reflect the broader systemic challenges posed by TFGBV. Although trauma-informed frameworks often highlight “freeze” and “fawn” as responses to threat or harm, these concepts are not yet explicitly addressed in the existing literature on TFGBV coping strategies. Such reactions may occur, particularly in cases of severe psychological stress, but they may not manifest as overt behavioral strategies in digital spaces.

A recent scoping review by Sheikh and Rogers (2023) examined the prevalence, impacts, and help-seeking behavior of technology-facilitated sexual harassment and violence among women across low- and middle-income countries. While the review emphasizes the considerable psychological and social impact on the targets, it also identifies a gap in the current literature concerning the diverse coping strategies employed by women to navigate such harassment. Furthermore, the review does not specifically focus on politically active women but highlights that women in paid employment and those from minority groups face “disproportionately” more online harassment (Sheikh & Rogers, 2023, p. 11). The present study aims to address these gaps by examining the complex and individualized responses to TFGBV by politically active women with a focus on the professional and emotional dimensions of these responses, as well as the coping mechanisms employed.

## Methods

### Search Strategy and Selection Criteria

We conducted this systematic review in line with the preferred reporting items for systematic reviews and meta-analyses (PRISMA) guidelines and followed a pre-registered protocol (*PROSPERO 2023 CRD42023453183*) to identify studies examining the multi-layered reactions of politically active women to TFGBV. For this review, we defined politically active women as individuals who engage in public-facing political roles or political activities that involve a significant level of public visibility and influence. This includes politicians currently in office or running for election, journalists, activists, human rights defenders, and social leaders who engage in public appearances or discourse as part of their professional or activist roles. We excluded individuals who merely express opinions on political topics online without holding formal roles or responsibilities in political or public spheres. We focused on studies involving politically active women aged 18 years and above and excluded those that focused on men or adolescent women. This age threshold was selected due to the fact that most political professions and roles have a minimum age requirement, typically set at 18 or above. Furthermore, much of the literature concerning adolescents predominantly addresses issues such as cyberbullying, which fall outside the scope of our investigation into political engagement and activism. Studies that included both genders were only included if the results were disaggregated by gender; studies that did not report separate results for women, even after contacting the author, were excluded.

TFGBV was broadly defined based on the existing literature as any form of violence committed through the use of information and communications technologies (ICTs). This includes psychological violence, such as threats of or intimidation with physical or sexual violence intended to cause fear, mobbing, trolling, or spamming. Sexual violence encompasses acts such as the non-consensual distribution of sexual images, defamation based on sexuality or sexual behavior, and rape threats. Economic violence refers not only to direct financial abuse—such as hacking bank accounts or coercing women into investing in additional security measures for themselves or their families—but also to indirect impacts like productivity losses, reputational harm, and career disruption stemming from online harassment. Physical violence includes digitally mediated threats such as the use of technology to track or monitor an individual with the intent to commit an assault, cyberstalking, and incitement to physical harm, resulting in changes to physical safety measures, such as increased offline security, hypervigilance, or relocation to another home due to fear of assault. Political, symbolic, and reputational violence includes impersonation intended to damage credibility or reputation, and the distribution of false or defamatory information aimed at discrediting women in political and public spheres. The studies selected for inclusion had a focus on TFGBV that was specifically perpetrated through digital tools, such as mobile phones, social media platforms, GPS technologies, or email (see OSF repository for full search string). We excluded studies that did not disaggregate results between online and offline violence, as our review focused exclusively on the impacts of violence facilitated through digital means.

To be eligible, studies also needed to examine at least one of the following outcomes: psychological and emotional responses to TFGBV, political or professional consequences of TFGBV, or coping mechanisms employed by women facing such violence. Psychological outcomes included reactions such as feelings of embarrassment, anxiety, psychological distress, depression, low self-esteem, post-traumatic stress, or concerns for personal or family safety. Political and professional outcomes were defined as changes in the frequency or nature of online or offline appearances, alterations to political communication (content, tone, or frequency), productivity losses, or withdrawal from political engagement altogether. Finally, coping mechanisms were categorized as strategies aimed at mitigating the effects of TFGBV, including increasing online or offline security, engaging in self-care, reporting abuse to platforms or authorities, or seeking social or professional support.

Both quantitative and qualitative studies were considered without any restrictions on research design or geographic context. Only studies published in English from January 1, 2000, onward were included, as this timeframe captures the rise of ICTs and the increased prevalence of TFGBV.

We searched the databases Scopus, Web of Science, APA PsycArticles, Social Science Citation Index, Applied Social Science Index and Abstracts, and PubMed between October 11 and 12, 2023, with a search update implemented on September 4, 2024. Additionally, gray literature was retrieved from the World Bank Open Knowledge Repository, National Democratic Institute, Amnesty International, Plan International, InternetLab, UNWomen, and UNESCO. Further, we used the AI tool *connected.papers* to identify additional resources. Lastly, we checked the reference lists of all included papers and reached out to all respective authors for potential additional relevant work. We used English search terms only to set up the search string, which was designed to reflect a wide range of gender-based violence committed by the use of ICTs. The database and gray literature search string are depicted in the Supplemental Material.

After eliminating duplicates, 100 titles and abstracts were independently screened by all reviewers to ensure consistency in applying the eligibility criteria based on the *Title and Abstract Inclusion Guide* (see Supplemental Material). Consequently, 10% of the remaining records (1,594 studies) were double-screened, yielding an inter-rater reliability (Cohen’s kappa) of .79, indicating substantial agreement. The remaining titles and abstracts (14,341 studies) were distributed and screened by one of the reviewers (see Supplemental Figure S1). Any discrepancies were resolved through joint discussions until a consensus was reached.

### Data Analysis

Data from included studies were entered into a piloted data extraction form to capture relevant information on (a) the country of the study, (b) the research methodology employed in the study, (c) the sample size, (d) information on the type of political activity the sample was pursuing, (e) the average age of study participants, and (f) any reported intersectionality characteristic of the study participants such as ethnicity, age, sexual orientation, or feminist views. Supplemental Table S3 captures the three outcome variables of interest, namely (g) psychological and emotional consequences to TFGBV, (h) professional and political consequences of TFGBV, and (i) coping mechanisms employed by targets of TFGBV. We categorized the reactions and coping mechanisms of the targets based on the predefined and consistently updated data-extraction guideline (see Supplemental Material). For this, we build on 19 broad categories of psychological reactions, 12 categories of political reactions, and 24 categories of coping mechanisms developed prior to the coding process based on existing literature (see Supplemental Table S2). These categories were designed to capture the range of reactions and responses documented in the studies. The extraction process was iterative and involved revisiting the predefined categories to ensure they accurately reflected the data. This back-and-forth approach allowed for adjustments, such as refining subcategories or identifying new ones, ensuring a comprehensive and accurate analysis of all relevant information.

One review author carried out the data extraction, 37% studies were double-extracted by another review author, and the remaining 63% were checked by another review author. Ambiguities were flagged and resolved by consent. Conducting a quantitative meta-analysis was not deemed appropriate, given that half of the included studies were based on qualitative work.

### Risk of Bias Appraisal

One reviewer assessed the study quality and risk of bias of the selected papers using validated tools specific to each study type. The quality of cross-sectional quantitative and qualitative studies was assessed with the Joanna Briggs Institute (JBI) tool (respectively, Lockwood et al., 2015; Moola et al., 2020). For mixed-methods studies, both tools were applied. A second reviewer verified half of these ratings. Ambiguities were flagged and resolved by consent.

## Results

**Figure 1.**
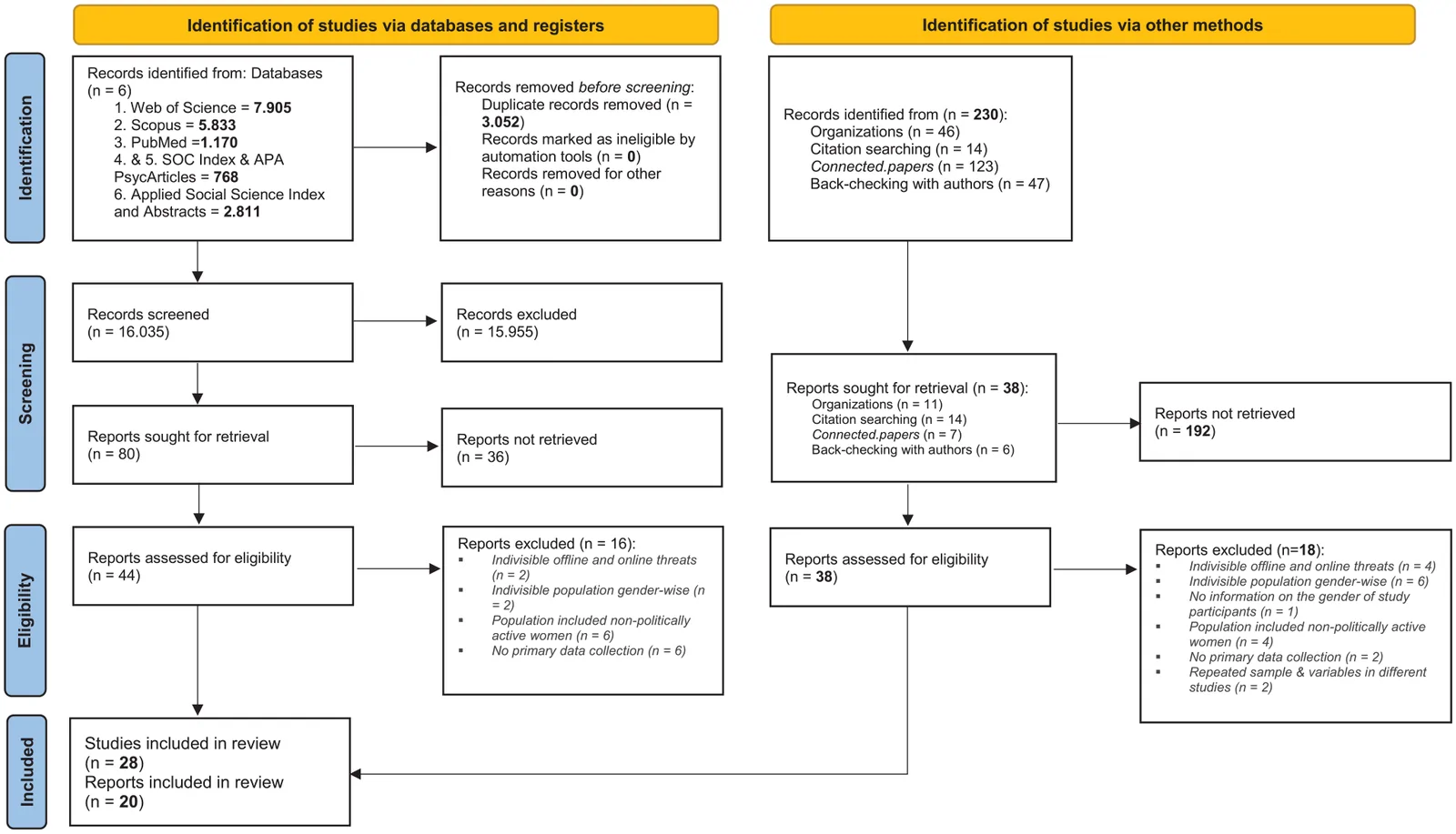
Study selection PRISMA 2020. *Note.* PRISMA = preferred reporting items for systematic reviews and meta-analyses.

Our database search returned 16,305 studies, and we identified 230 additional records from other sources. Following the title and abstract screening, the remaining full-text screening was conducted for 80 studies, with a total of 44 studies assessed for eligibility. Of those, 28 were included in the review. In the gray literature search, 38 reports were assessed for eligibility from which 20 reports were included. The final number of included studies was 48 (see Figure 1).

Table 1 presents the characteristics of the included studies (see Supplemental Table S3 for detailed subcategories of outcome variables reported by each included study).

**Table 1.** Characteristics of Included Studies.

Study Reference	Publication Type	Country of Study	Methodological Approach	Sample Size	Political Activity Study Participants	Average Age Study Participant	Intersectionality Characterstic(s) Study Participants
Carlson and Witt (2020)	Journal article	United States of America	Quantitative online survey	141	Journalist	No information	No information
Nelson (2022)	Journal article	United States of America	Qualitative interviews	22 female interviewees out of 37	Journalist	No information	Ethnicity
Zviyita and Mare (2023)	Journal article	Namibia	Qualitative interviews	12	Journalist	≈30.58	Ethnicity
Sampaio-Dias et al. (2023)	Journal article	Portugal	Qualitative interviews	25	Journalist	≈40.32	Age
Chen et al. (2018)	Journal article	Various	Qualitative interviews	75	Journalist	34	Ethnicity
Walulya and Selnes (2023)	Journal article	Uganda	Qualitative interviews	10	Journalist	No information	No information
Wagner (2022)	Journal article	Canada	Qualitative interviews	51 female interviewees out of 101	Politician	No information	Sexual orientation
Phillips et al. (2023)	Journal article	Australia	Quantitative online survey	26 female survey respondents out of 36	Politician	No information	No information
Pain and Chen (2019)	Journal article	Taiwan	Qualitative interviews	25	Journalist	No information	No information
Koirala (2020)	Journal article	Nepal	Qualitative interviews	48	Journalist	≈24.04	No information
Goyal et al. (2022)	Conference proceeding	Various	Qualitative interviews and focus group discussions	13 female interviewees out of 18, and FGD with 9	Activists & Journalists	≈38.8	No information
Kempton and Connolly-Ahern (2022)	Journal article	United States of America	Mixed-methods	11 female respondents out of 20	Journalist	No information	No information
Erikson et al. (2021)	Journal article	Sweden	Mixed-methods	126 female survey respondents, of 287; 28 female interviewees of 48	Politician	≈47.6	No information
Lee and Park (2023)	Journal article	Republic of Korea	Quantitative online survey	200 female survey respondents out of 404	Journalist	No information	No information
Stahel and Schoen (2020)	Journal article	Switzerland	Quantitative online survey	223 female survey respondents out of 637	Journalist	≈43.5	No information
Article 19 (2021)	Report	Various	Qualitative interviews	7	Journalist	No information	No information
Sarikakis et al. (2021)	Journal article	Austria	Qualitative interviews	9	Journalist	No information	Ethnicity
Binns (2017)	Journal article	United Kingdom	Mixed-methods	117 female survey respondents out of 267, and 1 female interviewee out of 4	Journalist	No information	No information
Bauschke and Jäckle (2023)	Journal article	Germany	Mixed-methods	31 female survey respondents out of 373	Journalist	No information	No information
Eckert (2017)	Journal article	Various	Qualitative interviews	109	Activist	≈38.11	Feminist views
García and Sequera (2021)	Report	Paraguay	Qualitative interviews	5	Activist	No information	No information
Sequera and Acuña (2023)	Report	Paraguay	Mixed-methods	107 female survey respondents, and 64 interviewees	Journalist	≈26.69	No information
Jane (2017)	Book chapter	Australia	Qualitative interviews	32	Activist	No information	No information
Kim and Shin (2022)	Journal article	Republic of Korea	Qualitative interviews and analysis of self-reflective articles	5 female interviewees out of 10	Journalist	No information	No information
Tandoc et al. (2023)	Journal article	Philippines	Qualitative interviews	8	Journalist	No information	No information
Kundu et al. (2021)	Book chapter	Bangladesh	Qualitative interviews	5	Journalist	No information	No information
Miller and Lewis (2020)	Journal article	United States of America	Qualitative interviews	19	Journalist	No information	Age
Uwalaka and Amadi (2023)	Journal article	Nigeria	Qualitative interviews	16 female interviewees out of 30	Journalist	No information	No information
Adams (2018)	Journal article	Various	Quantitative online survey	102	Journalist	No information	No information
Lewis et al. (2016)	Journal article	United Kingdom	Mixed-methods	226 female survey respondents, and 17 in-depth interview	Activist	≈37.58	Feminist views
Holton et al. (2021)	Journal article	United States of America	Qualitative interviews	14 female interviewees out of 31	Journalist	No information	No information
Akhtar and Morrison (2019)	Journal article	United Kingdom	Quantitative online survey	59 female survey respondents out of 154	Politician	No information	No information
Lekic-Subasic (2023)	Book chapter	Various	Mixed-methods	30 survey respondents and 5 interviewees	Journalist	No information	No information
Jovanovska (2022)	Report	North Macedonia	Mixed-methods	103	Journalist	≈39.93	No information
Deavours et al. (2022)	Journal article	United States of America	Qualitative interviews	13 female inerviewees out of 24	Journalist	No information	No information
Sobieraj (2021)	Book	Various	Qualitative interviews	52	Activists & Journalists	No information	No information
Ferrier (2018)	Report	Various	Mixed-methods	597 female survey respondents and 25 interviewees	Journalist	≈37.11	Age
Carson et al. *(*2022)	Journal article	Australia	Mixed-methods	205 female survey respondents out of 427, and 10 female interviewees	Politician	≈34.4 (from interview participants)	No information
Harlow et al. *(2023)*	Journal article	Various	Focus group discussions	11 female interviewees out of 20	Journalist	No information	No information
Barão da Silva et al. (2022)	Journal article	Brazil	Qualitative interviews	31	Journalist	39	No information
Kantola and Harju (2023)	Journal article	Finland	Qualitative interviews	16 female interviewees out of 22	Journalist	No information	No information
Posetti and Shabbir (2022)	Book	Various	Mixed-methods	714 survey respondents, and 184 interviewees	Journalist	No information	No information
Lewis et al. (2020)	Journal article	United States of America	Quantitative online survey	236 female survey respondents out of 559	Journalist	No information	Age
Celuch et al. (2023)	Journal article	Finland	Quantitative online survey	397 female survey respondents out of 695	Journalist	No information	No information
Celuch et al. (2022)	Journal article	Finland	Quantitative online survey	228 female survey respondents out of 510	Politician	≈52.7	No information
Smith (2019)	Book chapter	United Kingdom	Qualitative interviews and focus group discussions	26	Activist	No information	Feminist views
Baloch et al. *(2025)*	Journal article	Pakistan	Qualitative interviews	9	Journalist	No information	No information
Every-Palmer et al. (2024)	Journal article	New Zealand	Quantitative online survey	34 female survey respondents out of 54	Politician	No information	No information

**Figure 2.**
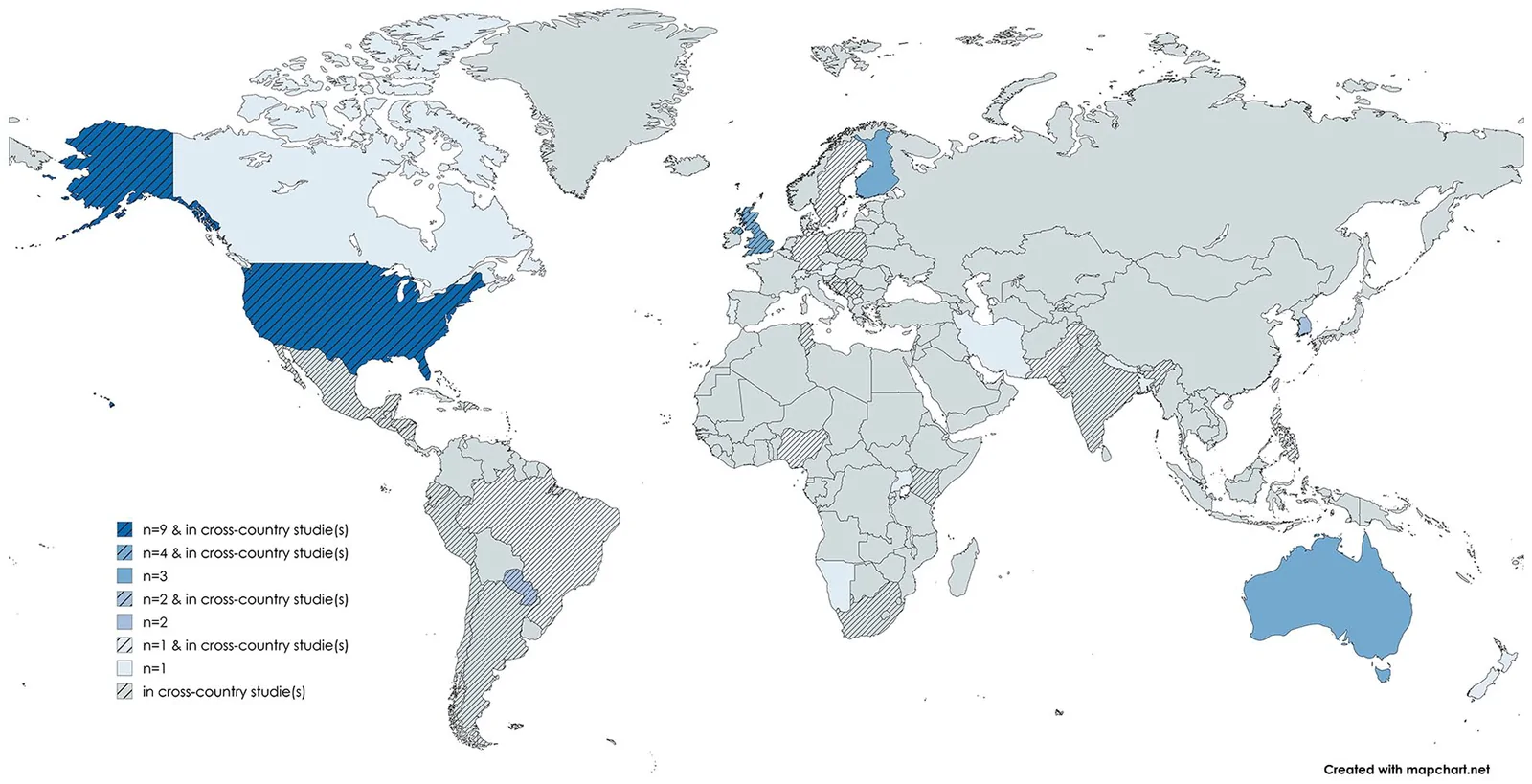
Geographical distribution of included studies. *Note*: Adams (2018), Ferrier (2018), Goyal et al. (2022) and Sobieraj (2021) were not included due to incomplete/unobtainable information on the country under study.

The included studies span various regions, with the highest number conducted in the United States (*n* = 9). Another notable concentration of research was found in the United Kingdom, contributing four studies, as well as Australia and Finland, each of which was represented by three studies. A small number of studies (*n* = 2) originated from South Korea and Paraguay. Ten studies also adopted a cross-country focus, further extending the geographical scope by examining data across multiple regions. The overall distribution highlights the strong focus on Western countries, particularly North America and Europe, with fewer studies from other regions, such as Africa and Asia (Figure 2).

**Figure 3.**
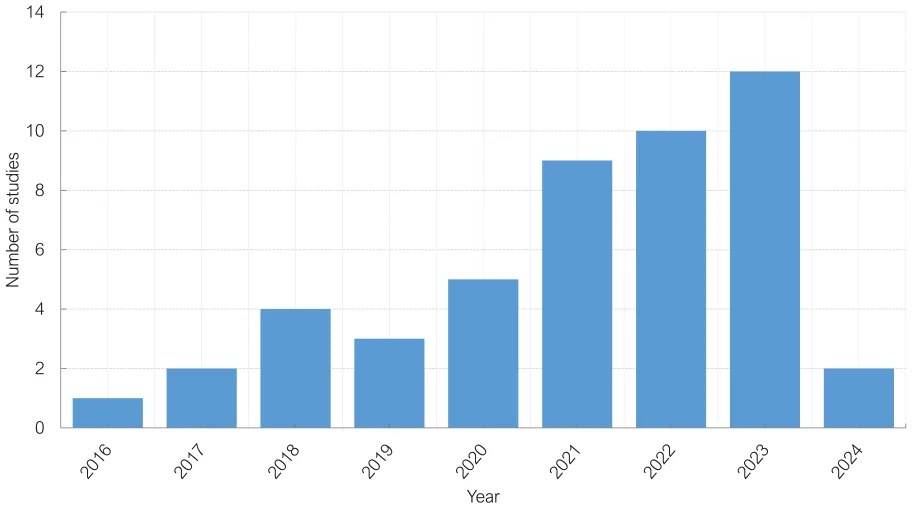
Distribution of studies per year.

Figure 3 demonstrates a clear upward trend in the number of studies published on the topic between 2016 and 2024. The number of studies reached a peak in 2023, with 12 published, indicating a growing scholarly focus on the issue.

Of 48 studies, 27 (56%) presented primary data collected through qualitative interviews and/or focus group discussions. Ten studies (20%) utilized data from quantitative online surveys, while the remaining 11 (22%) employed mixed methods designs, integrating both qualitative and quantitative approaches.

TFGBV in its various forms affected politically active women across a range of occupations: the majority of studies (34 out of 48 studies, 70%) focused on journalists. Seven studies (14%) examined the experiences of politicians, while five studies (10%) investigated TFGBV against activists. Two studies (4%) combined both activists and journalists in their analysis.

In terms of publication type, 36 out of 48 studies (75%) were published as peer-reviewed journal articles. Five studies (10%) appeared as reports, while 4 (8%) were included as book chapters. Two studies (4%) were published as books, and one study (2%) was presented as a conference proceeding (see Table 1).

### Psychological and Political Consequences of TFGBV and Coping Mechanisms

#### Psychological Consequences

**Figure 4.**
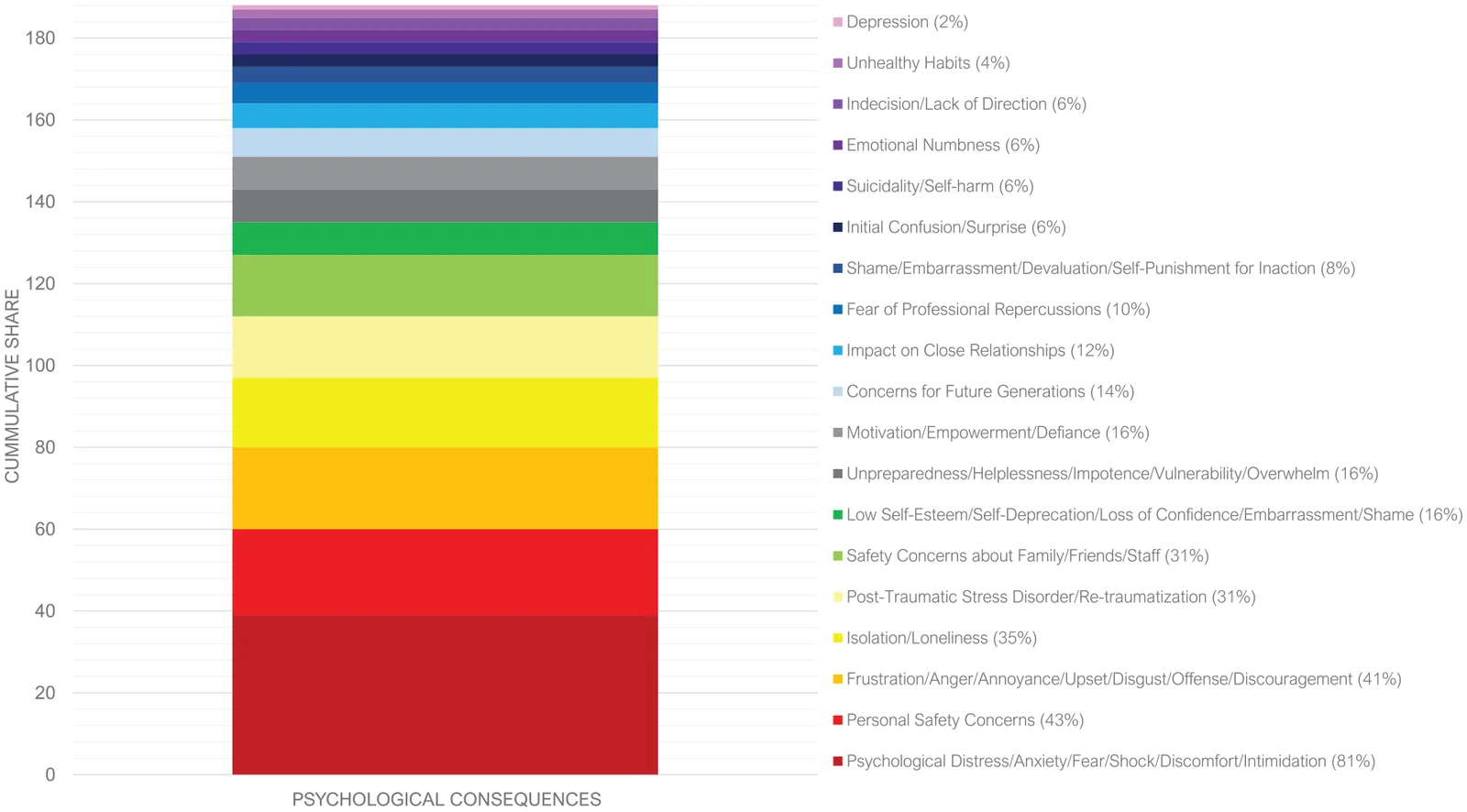
Psychological consequences. *Note.* The percentages represent the proportion of studies that address each subcategory.

The analysis of psychological consequences of TFGBV reveals significant emotional and mental distress among politically active women. Among the 48 included studies, 39 (81%) identified psychological distress, anxiety, fear, and related emotions as prominent outcomes (see Figure 4). These reactions are manifested in various forms, ranging from panic attacks and heightened anxiety to a deep sense of discomfort and intimidation. One activist described how this harassment “*fundamentally changed who I am and how I relate to the world*,” which led to panic attacks and a diagnosis of anxiety and depression (Sobieraj, 2021, p. 113). Another journalist explained how the online attacks “make me feel lesser than human” (Carlson & Witt, 2020, p. 9). In a study focused on politicians, 33% (*n* = 26) identified mental or emotional stress as a direct consequence of online harassment (Phillips et al., 2023, p. 15), and Stahel and Schoen (2020, p. 14) found that being a woman significantly increased stress due to received online harassment by 1.68 units, with stress defined as emotional upset or feeling frightened. Lewis et al. (2016, p. 12) found that 36% (*n* = 226) of surveyed activists affirmed that, “I was upset, and it had a significant impact [...].” Similarly, 42% (*n* = 226) of journalists in the same study reported being “worried” after the most recent incident, with stress and anxiety being frequently mentioned as consequences (Lewis et al., 2016, p. 14). Jovanovska (2022, p. 26) also highlighted that 46% (*n* = 103) of participating journalists reported feeling uncomfortable following incidents of online harassment. One activist described the pervasive fear she experienced, stating: “There’s this constant electric current of fear running through everything in your professional life” (Sobieraj, 2021, p. 30).

Furthermore, 15 (31%) studies highlighted how online harassment can serve as a “trigger” for re-traumatization, especially for women with previous experiences of violence. For example, in one included study, 26% (*n* = 226) of journalists indicated that their experiences with harassment were traumatic, stating: “I keep thinking about it even though I don’t want to” (Lewis et al., 2016, p. 12). Likewise, Sobieraj (2021) highlighted the psychological toll of TFGBV with one writer describing how she developed anxiety every time she saw her phone: “It just felt, I don’t know if it’s Pavlovian, but every time I saw my phone, I would have anxiety” (Sobieraj, 2021, p. 33). The physical effects of trauma were further emphasized in Ferrier’s (2018, p. 37) research, where 56% (*n* = 597) of respondents reported having strong physical reactions—such as heart pounding, trouble breathing, and sweating—when reminded of the stressful experience. Additionally, 53% (*n* = 597) mentioned actively avoiding internal reminders, including thoughts, feelings, or physical sensations (Ferrier, 2018, p. 37). One blogger described: “I had a nervous breakdown, where I literally thought there were people after me... I was convinced... this was my thinking” (Sobieraj, 2021, p. 114). This sense of long-term psychological deterioration is described by a journalist: “I have been living in a sense of constant fear that anything bad could happen” (Baloch et al., 2025, p. 7). Other studies corroborate the deep emotional impact of online harassment, with one journalist summarizing: “To be a victim of harassment is a traumatic experience” (Posetti & Shabbir, 2022, p. 37). Finally, the severity of trauma is reflected in the experiences of one activist who disclosed: “I was in the sort of complete trauma state of just red alert, fight or flight, didn’t sleep all night, for many nights” (Smith, 2019, p. 17). This ongoing sense of threat, combined with the memory of past abuse, underscores the enduring and compounding effects of TFGBV, making it not just a momentary challenge but a source of prolonged mental health issues.

The fear induced by online violence was not confined to the digital realm. Twenty-one studies (43%) highlighted how online threats spurred concerns for personal safety and 15 studies (31%) referred to concerns regarding the safety of loved ones. In one included study, 69% (*n* = 34) of female politicians expressed fear for their safety, and 46% (*n* = 34) indicated that they felt unsafe in their own homes (Every-Palmer et al., 2024, p. 7). For example, one woman stated: “The negative interactions make me second-guess not just my work, but my safety and the safety of those around me” (Carlson & Witt, 2020, p. 9). Another politician noted: “They [the online attacks] can be frightening. You assume they’re just words, but it’s hard to know for sure that the person won’t make an effort to seriously injure you at some point” (Carson et al., 2022, p. 9).

In addition to fear, the impact on family members was also reported in 6 (12%) studies, with one woman stating: “My husband was physically attacked by someone launching at me in public” (Every-Palmer et al., 2024, p. 7). Concerns about the safety of family and friends were further reflected in Ferrier’s (2018, p. 26) study, where 39% (*n* = 597) of journalists indicated that they were “concerned about threats to family and friends” as a result of their own experiences with online harassment.

Feelings of self-doubt, self-criticism, and isolation also emerged as a result of online harassment. Seventeen studies (35%) indicated that women felt isolated, and eight studies (16%) reported feelings of shame or a loss of self-confidence in the face of these attacks. One study found that 14% (*n* = 226) of journalists reported “feeling embarrassment or shame” as a result of online harassment (Lewis et al., 2016, p. 14). As one journalist expressed it: “Sometimes, when you receive a lot of these comments, you tend to believe them. [...] With all these people saying these bad things, maybe I am a bad journalist” (Tandoc et al., 2023, p. 13). Another activist described the psychological impact of feeling devalued by online comments: “It does take a hit to your self-esteem, and you start thinking, ‘Maybe I didn’t have anything worthwhile to say. Maybe I shouldn’t have said anything at all’” (Sobieraj, 2021, p. 76).

In response to these psychological pressures, some of the studies revealed unhealthy psychological reactions. Two studies (4%) specifically highlighted behaviors such as shopping or gaming addiction as a way of managing the emotional toll of harassment. One journalist confessed, “I became very depressed. I had a shopping addiction. I was coping with it in different ways. I had a computer gaming addiction” (Sobieraj, 2021, p. 97). Similarly, Holton et al. (2021, p. 9) observed that journalists reported behaviors such as “running, stress eating, and alcohol.” The strain of harassment also led to heightened emotional distress, with one journalist reflecting: “Like, I was definitely drinking a little too much, and moody, and upset, and unhappy. It sucked. It just sucked” (Sobieraj, 2021, p. 113).

Concerns about the impact of online harassment on future generations were raised in 7 of the 48 included studies (14%). Several politically active women expressed worry that the sustained digital abuse they experience may have a chilling effect on younger women considering activism or political careers. As one activist noted: “I worry, too, that [digital abuse] might be silencing—it might be [hurting] activism, because women don’t want to face the sexism online” (Sobieraj, 2021, p. 113). This apprehension was shared by other journalists who, while having developed refusal to be intimidated over time, worried about the effect on newcomers: “Those of us who lived through the times of other characteristics will have a thicker skin for that, but it is quite worrying for those who are just starting out” (García & Sequera, 2021, p. 27).

Despite the psychological toll of online harassment, some women exhibited a refusal to be intimidated in the face of harassment, using their experiences as motivation to continue their political engagement. In one study, 54% (*n* = 226) of activists reported feeling more determined about their political views, 33% (*n* = 226) felt motivated to continue participating in debates, and 34% (*n* = 226) felt motivated to do something about the received attacks (Lewis et al., 2016, p. 14). The same sense of defiance was captured in another study by a journalist who noted: “I have to do what I have to do, I have to say what I believe in” (Posetti & Shabbir, 2022, p. 81).

The complex and varied psychological responses highlight the profound emotional toll that TFGBV can have on politically active women. These responses range from psychological exhaustion and fear to refusal to be intimidated and determination, reflecting the deeply personal and context-dependent nature of such experiences.

#### Political Consequences

**Figure 5.**
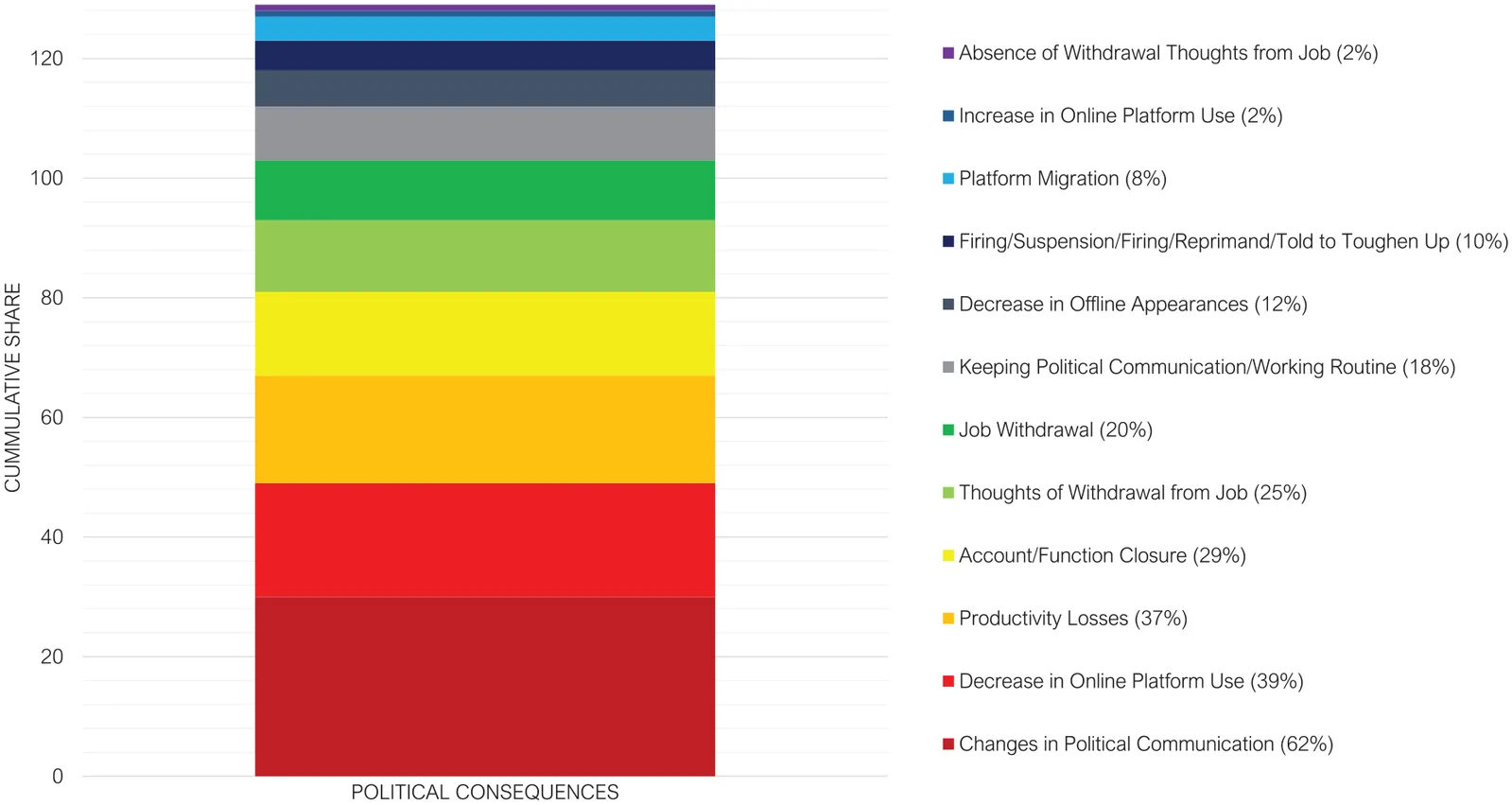
Political consequences *Note.* The percentages represent the proportion of studies that address each subcategory.

The political consequences of TFGBV are significant, with many women reporting changes in their public engagement and political communication. Of the 48 included studies, 30 (62%) documented alterations in the content, tone, or complexity of political messaging in direct response to experiences of TFGBV (see Figure 5). Women often adapted their communication to avoid further attacks, with one journalist stating: “I’ve held back arguments,” while others noted that they became “less vocal” on social media platforms (Adams, 2018, p. 8). Binns (2017, p. 11) found that 17% of journalists changed how they researched or wrote articles. One journalist from another included study explained: “I don’t feel if I’m going to talk about sexual harassment, I need to make a fool-proof argument about it and look up tons of specifics” (Sobieraj, 2021, p. 66). Lewis et al. (2016, p. 15) noted that 26% of journalists were “more cautious” in how they participated in discussions or which topics they chose to address. Some women refrained from discussing controversial topics altogether, as exemplified by one feminist blogger who chose not to write about charged issues like guns and abortion to avoid “opening up a can of worms” or another participant who “decided not to use the word ‘feminism’ on her blog to minimize misogynist comments” (Eckert, 2017, p. 14). Some included studies aimed to quantify this self-censorship. For example, in the study by Koirala (2020), 6% of journalists reported abstaining from controversial topics, while 4% dropped a news story following an incident of online harassment, and 6% avoided reporting on particular issues due to fear of abuse (pp. 7–8). Additionally, Ferrier (2018, p. 39) found that 37% (*n* = 597) of journalists actively avoided certain stories. The percentages across these studies may vary, but collectively, they highlight a substantial issue: even a relatively small percentage of women self-censoring or changing their work practices can lead to significant gaps in public discourse, particularly around controversial or critical issues.

A substantial proportion of women also opted to reduce their online presence. Nineteen studies (39%) reported a decrease in online platform use, and 14 studies (29%) documented women closing or abandoning their accounts to escape abuse. One journalist explained how she dropped her byline from her stories to avoid being targeted by trolls (Tandoc et al., 2023, p. 11). Quantitative evidence from various studies further underscores the extent to which the online presence of targeted women can be affected and reduced. Binns (2017, p. 11) reported that 13% (*n* = 45) of journalists stopped using a particular platform, while Ferrier (2018, p. 39) found that 5% (*n* = 597) of respondents went so far as to delete their profiles or accounts. Similarly, 46% (*n* = 17) of journalists surveyed in the study by Sequera and Acuña (2023, p. 22) reported temporarily suspending their profiles on social media networks to avoid further attacks. In addition, Posetti and Shabbir (2022, p. 82) noted that 11% (*n* = 79) of journalists had permanently withdrawn from certain online communities. The decision to step back or withdraw from online spaces represents a troubling shift, as it ultimately leads to a more constrained and homogeneous public sphere where fewer voices are heard.

Productivity losses were another common outcome, with 18 studies (37%) highlighting how the emotional and psychological toll of TFGBV impeded women’s ability to work effectively. One woman described how sexist abuse prevented her from meeting informants, thus hindering her journalistic work (Adams, 2018, p. 10). Another noted how participating in her profession required wading through “filth,” which not only took a mental toll but also extended the time needed to complete basic tasks: “It takes longer for women to just do the basic function of participating, and that’s even when you take out fear for one’s safety, or the psychological effects that it might have on you, or the stress it causes, and what that might do to your body, and how you might need to rest up more” (Sobieraj, 2021, p. 112). Such cognitive load exacerbated the challenges of working in a hostile digital environment, forcing women to expend additional energy to maintain their professional roles: “And I can tell you that it occupies more brain space in women than it should because it’s one more thing we have to deal with. It sucks” (Sobieraj, 2021, p. 106). Quantitative data from various studies further underscore the impact of online harassment on professional productivity and reputational damage. For example, Phillips et al. (2023, p. 15) found that 14% (*n* = 26) of politicians identified damage to their reputations as one of the primary problems they experienced due to harassment. Adams (2018, p. 9) reported that 4% of journalists explicitly commented that their careers had been damaged, with one journalist stating, “it hurt my career.” Further illustrating the toll on productivity, Lewis et al. (2016, p. 14) cited one activist who took sick leave, explaining: “It made me substantially more sick and it took around 2 to 3 months before I was back to a useful level of ‘health’ again.” In addition, Akhtar and Morrison (2019, p. 4) reported that 66% (*n* = 59) of the surveyed politicians identified damage to their reputation, 59% (*n* = 59) noted professional problems at work, and 37% (*n* = 59) stated they lost time at work as a result of the harassment. These findings highlight how online harassment, even if not always immediately visible, can have far-reaching impacts on women’s professional lives, reputations, and overall productivity. Despite the relatively small percentages in some studies, the cumulative effect of these experiences remains concerning, as even a modest impact on professional life can lead to longer-term career setbacks for politically active women.

In the most extreme cases, the harassment led to thoughts of withdrawal from political roles or careers. Twelve studies (25%) noted that women considered leaving their jobs, and 10 studies (20%) documented actual cases of job withdrawal. One journalist remarked: “I’ve often thought of getting out of this business because of negative online interactions. Life is too short” (Carlson & Witt, 2020, p. 9). Quantitative data from Lee and Park (2023, pp. 13–14) highlight the causal link between online harassment, psychological trauma, and the intention of female journalists (*n* = 200) to leave the profession. The study found that online harassment had a significant effect on both the intention to leave (β = .19, *p* < .01) and levels of trauma (β = .43, *p* < .001), whereby trauma itself also significantly influenced the intention to leave (β = .21, *p* < .01).

Despite these negative outcomes, nine studies (18%) reported that the politically active women maintained their political communication routines in the face of harassment. These women chose to continue their work, often framing their experiences as a call to “toughen up” or resist the pressure to withdraw. Nevertheless, the overall trend indicates that TFGBV significantly alters how politically active women engage with their audiences, impacting not only their personal well-being but also their professional productivity (37%) and public visibility (12%).

#### Coping Mechanisms

**Figure 6.**
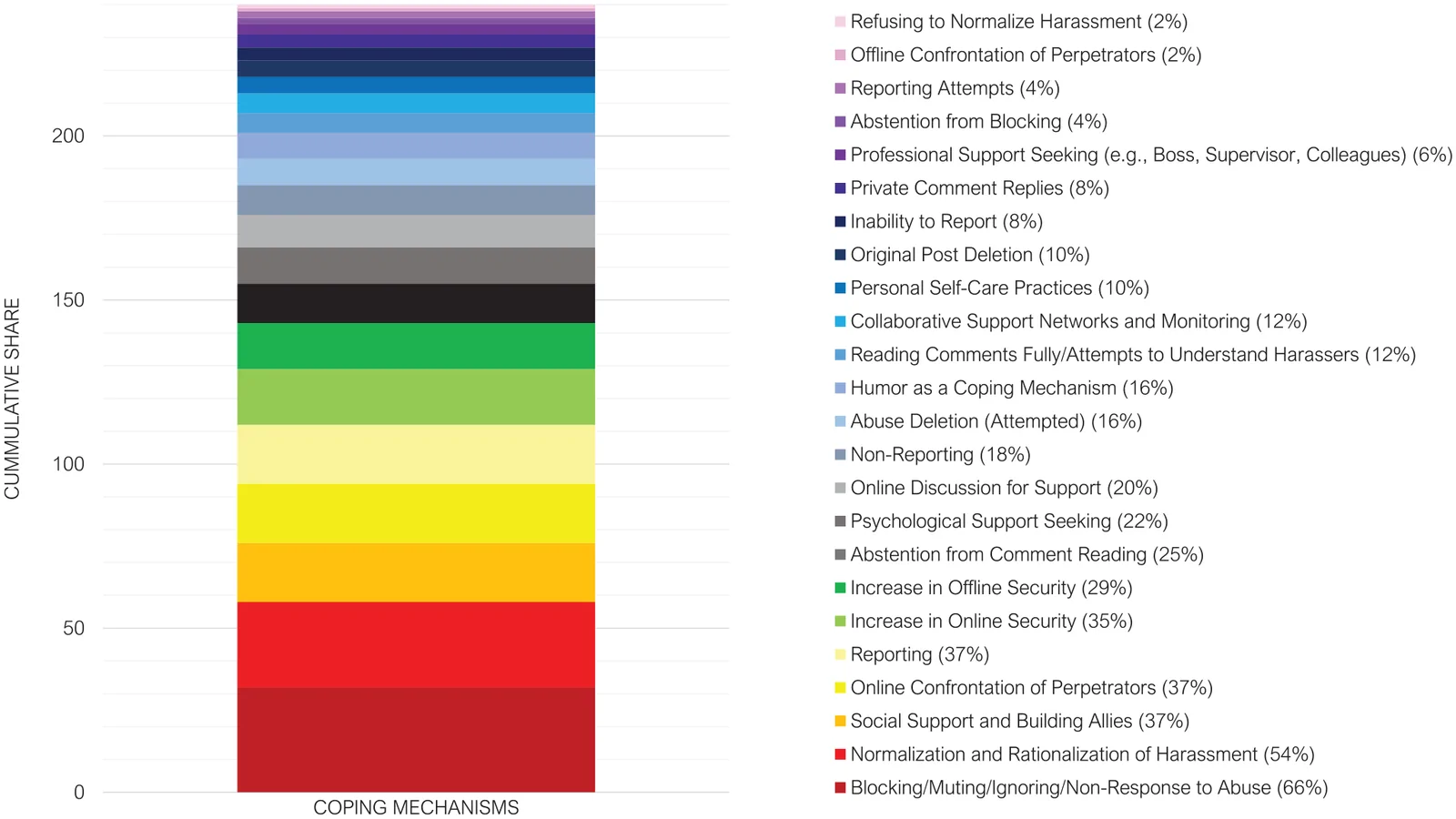
Coping mechanisms. *Note.* The percentages represent the proportion of studies that address each subcategory.

Politically active women employ a wide range of coping mechanisms in response to TFGBV. The most frequently reported coping strategy was blocking, muting, ignoring, or non-response to abuse, identified in 32 (66%) of the studies (see Figure 6). Women often choose to ignore harassers or block them on digital platforms to prevent further interaction, thus minimizing the psychological toll of direct engagement. One journalist described this approach, stating: “I don’t retaliate against bullies on social media. For me, one thing I do is block them and move on” (Zviyita & Mare, 2023, p. 11). Also, across the studies collecting quantitative evidence, blocking and ignoring harassers were consistently reported as common coping mechanisms. Ferrier (2018, p. 38) found that 54% (*n* = 587) of journalists blocked the offender as a way of coping with the abuse. Sixty-two percent (*n* = 48) of journalists in another study mentioned ignoring the abuse altogether (Koirala, 2020, p. 7), while 66% (*n* = 88) of journalists from another study reported blocking the offending users (Binns, 2017, p. 11). Similarly, Bauschke and Jäckle (2023) found that 62% (*n* = 16) of journalists chose not to react to or engage with the harassers. In another study, 57% (*n* = 30) of the journalists opted to ignore the abuser, and 50% (*n* = 30) blocked the abuser (Lekic-Subasic, 2023, pp. 18–22).

Twelve studies (25%) indicated that the targets abstained from reading the comments under their posts. Binns (2017, p. 11) found that 64% (*n* = 45) of journalists reported “stopping reading the comments under my articles,” while 47% (*n* = 30) of journalists in a separate study also “stopped reading online comments” as a coping mechanism (Lekic-Subasic, 2023, p. 18). Another journalist said: “I never, ever, ever read the comments on articles that I write. Because they’re garbage. . . Comments are just a cesspool of anger and hatred” (Sobieraj, 2021, p. 121). As another activist reflected: “I’ve not read a comment on anything I’ve written in at least two, two and a half years” (Sobieraj, 2021, p. 122).

Normalization of TFGBV was the second most common response to harassment, a strategy where women downplayed the severity of the abuse, reported in 26 of the studies (54%). For instance, Eckert (2017, p. 13) found that 17% (*n* = 80) of activists stated that they “got used to it” over time. One woman explained this mindset: “I have worked hard to cultivate an attitude of shoulder-shrugging callousness. Because what else am I going to do? I can’t go through [life] depressed” (Sobieraj, 2021, p. 84). This internalized trivialization was reflected in the narratives of women who worked hard to suppress the emotional toll of the attacks, often portraying themselves as unaffected to avoid victimization or inconvenience. This attitude was further supported by a journalist who refrained from discussing her experiences, stating: “I’ve never told my colleagues about it because I just always thought, ‘It’s probably not that serious, or they probably have it worse... Someone fought me on Facebook.’ It doesn’t sound like a real problem” (Tandoc et al., 2023, p. 12).

This normalization of harassment is often linked to the perception that enduring abuse is an inherent part of being a public figure. One journalist noted: “As long as you are in the profession, you have to tolerate it” (Kundu et al., 2021, p. 16), while another highlighted the need to develop a “thick skin” to cope with the abuse: “Look, I kind of try to overcome these things. Before, I would be sick with what I was told. But we must leave it behind; otherwise, we don’t live” (Sampaio-Dias et al., 2023, p. 13). The problematic comparison with male resilience was also evident, as many women felt compelled to adopt coping strategies traditionally associated with “masculine strength.” Corroborating this, one reporter observed that “the major strategy was to be strong-hearted like a man” (Koirala, 2020, p. 8). The emotional labor involved in minimizing or normalizing the harassment represents a significant aspect of the coping process, as women are forced to navigate the psychological impacts while simultaneously managing the expectations of their professional roles.

Reporting abuse was the third most common strategy, mentioned in 18 studies (37%). Women sought recourse by reporting harassers to platform moderators, authorities, or their employers. Several studies provided detailed insights into the prevalence of reporting, which varied between 3% and 43%. Jovanosvska (2022, p. 29) reported that 32% (*n* = 103) of journalists took action by reporting the abuse to the editorial staff. Similarly, Lewis et al. (2016, p. 14) noted that 43% (*n* = 226) of the journalists talked to formal contacts, such as the police, internet service providers, or platform moderators. Lekic-Subasic (2023, p. 18) found that 16% (*n* = 30) of the journalists reported the abuse to the police. Ferrier (2018, p. 38) revealed that 28% (*n* = 597) of respondents reported harassment, threats, or attacks on the online platform, while 14% took the step of reporting the abuse to the police. Celuch et al. (2023, p. 22) provided additional data, with 14% (*n* = 152) of respondents reporting incidents to the police. However, non-reporting was also prevalent, documented in nine studies (18%), with many women expressing a lack of confidence in the effectiveness of reporting mechanisms or institutional support

Many women also sought social support and allies, identified in 18 (37%) of the included studies. They relied on networks of friends, family, and colleagues to share their experiences and receive emotional support. Journalists in particular even formed cross-national alliances to expose online harassment and protect themselves from further abuse. As one journalist stated: “The international alliances have been fundamental to be able to reveal what is happening here but also to protect us in this work” (Harlow et al., 2023, p. 12). Peer assistance, such as moderating social media accounts for each other, was another significant form of support (Kantola & Harju, 2023). As cited in one study: “We mobilise each other from different regions of the country and occasionally from beyond the country. We usually respond by calling the perpetrator out and otherwise engaging with these trolls so that you, as the victim, don’t feel you’re alone” (Posetti & Shabbir, 2022, pp. 69–70).

Seeking psychological support was also a critical coping mechanism, as reported in 11 (22%) of the studies. Furthermore, three studies (6%) reported that politically active women turned to professional support from supervisors or bosses highlighting that only a small number of affected women actively reached out for help. For instance, Jovanovska (2022, p. 27) found that 4% (*n* = 103) of journalists sought psychological support. In another study, 12% (*n* = 86) of journalists reported seeking medical or psychological help as a response to online harassment (Posetti & Shabbir, 2022, p. 78). Celuch et al. (2022, pp. 3, Appendix C) further noted that 8% of participants stated, “I turned to counselling.”

Active approaches like online confrontation of perpetrators were noted in 18 (37%) of the studies. Some women choose to directly address their harassers, either by responding publicly or threatening legal action. This approach allowed women to take matters into their own hands by discrediting or challenging the abuse in a public forum, often using social media to document incidents and fight back against the harassers. One example involved a journalist who traced harassing emails to an employee of a telecommunications company and threatened legal action unless the abuse stopped (E. Jane, 2018, p. 11). Another one involved: “[A] journalist [...] remembers replying to the people sending threatening messages, even calling them. [...] At some point, she even called the harasser’s wife, which put an end to harassment” (Kantola & Harju, 2023, p. 10). In contrast, offline confrontation of perpetrators was reported in only one study (2%).

Other women used humor as a coping mechanism encountered in eight studies (16%), finding ways to reframe the abuse in a less emotionally harmful manner. For instance, Eckert (2017, p. 13) found that 6% (*n* = 80) of activists reported using humor as a strategy to cope with online abuse. In another study, one journalist humorously responded to the flood of outraged comments she received: “One woman received so many outraged comments asking if she had no shame, that she and her interns plastered them up in the office on a ‘Wall of Shame’” (Binns, 2017, p. 10). Another example involved a journalist who responded to an insulting comment about her health by making light of it, saying: “I have become resistant to comments on articles. ‘Your uterus smells like it is rotting.’ The annual medical check-up shows that my uterus is healthy, so I think it is fine” (Kim & Shin, 2022, p. 8).

Personal self-care practices such as engaging in stress-relief exercises to manage the psychological strain were encountered in five studies (10%). For instance, one study noted that “participants indicated that they preferred dealing with such infringements at an individual level” (Zviyita & Mare, 2023, p. 11), a sentiment echoed by Koirala (2020, p. 8), who found that most journalists from Nepal attempted to address the issue of harassment personally. This tendency toward isolation was further reflected in findings by Sampaio-Dias et al. (2023), who observed that journalists often normalized their experiences or contextualized them within broader structural inequalities: “There is a problem for us journalists; we don’t talk about our problems ... We only report ... extreme cases when journalists are killed ... We don’t talk when journalists are victims of harassment...” (p. 12). The reluctance to seek external support was also influenced by cultural stigmas surrounding mental health, as highlighted by a participant from Kenya: “Seeking help for mental health here in Kenya is looked down [upon] ... So we find that people don’t seek help, they deal with it individually” (Posetti & Shabbir, 2022, p. 79). These findings suggest that applied coping mechanisms are also context-dependent, sometimes leading women to internalize or individually manage the psychological strain of online harassment rather than seek collective or professional support.

Finally, increasing online and offline security was a critical response to harassment, with 17 studies (35%) reporting enhanced online security measures and 14 studies (29%) increased offline security. In terms of online security, politically active women took various steps to protect their digital presence, including hiding personal information, changing passwords, and using anonymous identities or spam filters. Binns (2017, p. 11) reported that 28% (*n* = 45) of journalists had changed their profile pictures, while 12% (*n* = 597) of women in Ferrier’s (2018, p. 38) study altered their online identity by modifying their profile picture or name. For many, securing social media accounts became an essential defense mechanism against ongoing harassment. One journalist remarked: “[I have] lots of experience of being trolled—good thing I know how to secure my social media account” (Tandoc et al., 2023, p. 9). The necessity for constant vigilance also extended to regularly updating security settings, as the same study noted: “every now and then, they have to alert their editors and also change their passwords” (Tandoc et al., 2023, p. 14).

Similarly, 35% (*n* = 226) of the activists in Lewis et al.’s (2016, p. 16) study employed security measures such as blocking contacts or adjusting privacy settings. This sentiment was echoed by another activist, who expressed the need to remove public calendars from her website due to fear of potential attacks: “I used to have a calendar on my website, that I’m going to be at this conference or do this talk because this is what you do. You can meet people with whom you have something in common and have coffee. I don’t have a calendar anymore. I don’t know if the [attackers are] serious every once in a while when they say they want to blow my brains out or they want to kick my ass” (Sobieraj, 2021, p. 108).

Increased offline security was equally important. Some participants altered their daily routines to avoid potential threats, such as changing walking routes or avoiding traveling alone at night. Binns (2017, p. 11) found that 15% (*n* = 45) of journalists reported changing their behavior offline, such as walking home via a different route. Furthermore, 6% (*n* = 30) of journalists in Lekic-Subasic’s (2023, p. 20) stated that online harassment had led them to alter their offline behavior, such as choosing alternative routes home, while a more extreme case involved a participant who had to temporarily leave the country due to the severity of threats. Increased offline security seems to be especially relevant for politicians. Akhtar and Morrison (2019, p. 4) noted that 96% (*n* = 59) of politicians increased their security at home, with the same percentage taking additional security measures at work. One woman explained the extent of her offline security measures: “I had to protect myself by any means necessary, be it putting lighting at my house, making it look like it’s daylight at night, having cameras everywhere, having bodyguards... I even park differently, in case somebody tries to attack. I’m always looking over my shoulder” (Posetti & Shabbir, 2022, p. 94). The hyper-vigilance associated with public appearances was also noted, with one woman stating: “The first thing I do whenever I’m giving a public talk [. . .], I scan the room for threat. I try to figure out who the [threat] in the room might be and what they might say and what they might do” (Sobieraj, 2021, p. 70).

These security measures, both online and offline, require not only significant time investment but also impose an additional emotional burden on the women affected. For many, the constant threat of harassment shaped everyday decisions, influencing even their most personal choices, such as where they lived. As one journalist noted: “It has affected different considerations, like what type of home I live in, for example, never a ground-floor home, or anywhere where I won’t feel safe walking from my car to my front door” (Article 19, 2021, p. 7). These findings highlight the significant steps women have been forced to take to safeguard both their physical and digital environments in response to online harassment.

Overall, women’s responses vary from avoidance and self-preservation to confrontation and collective support, reflecting the multifaceted nature of the challenges they face in public and political life.

#### Intersectionality: Gender, Ethnicity, Sexuality and Age

Individual characteristics, including ethnicity, sexual orientation, age, and feminist views, may intensify the vulnerability of politically active women to TFGBV. Among the studies reviewed, 12 (25%) mentioned the influence of one or more intersectional characteristics on the experiences of politically active women with regard to TFGBV. These characteristics not only impact the nature of the abuse experienced by women but also influence the psychological and political consequences and the coping strategies they employ in response to it.

Several studies highlighted the compounded challenges faced by women of color and those from marginalized communities. As documented by Sarikakis et al. (2021), women of color frequently encounter harassment that targets both their gender and ethnicity, creating a dual-layered experience of hostility. To illustrate, one journalist emphasized: “It’s all about the colour of my skin, that’s what I get hate comments about. Being a woman is no problem. And I think that’s the reason why I can handle hate comments well, because I’m used to them. Since my childhood, I get hate in real life, the same hate I get online now” (Sarikakis et al., 2021, p. 10). This recurring experience of discrimination may contribute to the development of resilience, whereby some women become more tolerant of hostility and develop a stronger emotional defense.

Similarly, women who identify as LGBTQ+ often experience harassment that is not only gendered but also homophobic. As Wagner (2022, p. 12) observed, politicians of under-represented groups in Canadian politics frequently express heightened concerns about online harassment due to its intensely personal nature. This intersection between gender and sexual orientation intensifies the perceived severity of threats, affecting both their emotional well-being and political engagement. As one participant noted, their LGBTQ+ identity often becomes a focal point for harassment, merging personal and political attacks in ways that amplify psychological distress.

The types of abuse experienced by politically active women are influenced by their feminist identification, with many reporting intensified hostility due to their advocacy work. As illustrated by Jane (2017, p. 9), some activists were reluctant to republish abusive messages due to concerns about potential further backlash, despite admiring the courage of others who publicly shared their experiences. In contrast, some women, such as a feminist activist who participated in Eckert’s (2017) study, employed public platforms to expose abusive behavior and reclaim control over the narrative. This divergence highlights the impact of feminist views on coping responses, with some women adopting a cautious approach to managing their visibility, while others employ activism as a form of resistance.

Moreover, age appears to exert an influence on how politically active women interpret and respond to TFGBV, as evidenced by four of the studies. Sampaio-Dias et al. (2023) found that older women tended to minimize the gendered aspects of harassment, instead framing it as opposition to their professional roles. A senior journalist reflected, “I am not a man or a woman, I am a journalist” (Sampaio-Dias et al., 2023, p. 10). This assertion suggests that experience, frequently associated with age, influences how women perceive harassment. Some women regard it as an obstacle to their professional identity rather than a gender-related issue, which affects their coping mechanisms. Nevertheless, this refusal to be intimidated is not a universal phenomenon among women. As Posetti and Shabbir (2022) observe, women in the early stages of their careers are more likely to feel isolated and to encounter greater difficulties in managing online abuse: “I remember feeling upset and really intimidated, especially as I was still at the start of my career” (Posetti & Shabbir, 2022, p. 75). Furthermore, women who lack access to institutional support or adequate digital resources often find themselves isolated, relying on individual coping strategies such as the suppression of thoughts related to the abuse or connective practices like seeking support from peers, colleagues or (cross-national) allies (Harlow et al., 2023; Kantola & Harju, 2023; Posetti & Shabbir, 2022). This further emphasizes the unequal distribution of coping resources, whereby some women—particularly those with higher levels of self-efficacy or social capital—are better equipped to manage the harassment, while others encounter greater obstacles to maintaining their professional visibility.

Women from lower socioeconomic backgrounds or marginalized communities encounter distinctive challenges in management of TFGBV. Sobieraj (2021) posits that women from disadvantaged backgrounds frequently develop a tolerance to hostility as a result of prior exposure to discrimination. In this context, one participant commented: “You grow up in a rough neighbourhood or get sexually harassed young... nothing that was happening [online] was out of the ordinary” (Sobieraj, 2021, p. 94). Another commented: “I grew up in the hood. I know when it’s really dangerous. And while the online insults and racial epithets are hurtful and they suck, most of the time, nine times out of ten, these fools aren’t going to do anything else” (Sobieraj, 2021, p. 95). However, resilience developed in the context of adverse conditions may result in these women becoming isolated, as they may lack access to institutional support or professional networks, leaving them reliant on individual strategies and peer support (Harlow et al., 2023; Kantola & Harju, 2023).

These findings illustrate the importance of intersectionality in shaping the psychological and political impact of TFGBV, as well as in influencing the resources women can access for coping.

#### Risk of Bias Appraisal

Supplemental Table S3 presents the quality ratings based on the JBI tool that was applied to assess the included quantitative cross-sectional studies. The main limitation of the cross-sectional studies was the lack of consideration for potential confounding factors, with only 4 of the 10 studies explicitly identifying or accounting for these variables. Firstly, Lee and Park (2023) addressed potential confounding factors, including age, work position, gender and political affiliation in their analysis. Secondly, Stahel and Schoen (2020) incorporated variables pertaining to demographics and media reach. Thirdly, Lewis (2020)controlled for a number of variables, including age, race, education, income, media type, and social media use, in order to isolate the effect of harassment on journalists’ perceptions. Lastly, Celuch et al. (2022) employed logistic regression analysis to account for factors such as minority status, personality traits, and social media use, thereby ensuring a comprehensive approach to analyzing responses to harassment. The remaining six studies did not address confounders that could affect the relationships being measured, which may have limited the interpretability of the results.

Apart from this, the outcome measures employed in half of the studies were downgraded due to the use of self-reported outcomes and the absence of a previously validated scale to assess the psychological or political consequences of TFGBV or coping mechanisms. Although this approach effectively captures personal insights, it is limited in terms of validity and reliability due to its dependence on participants’ subjective interpretations. For example, Phillips et al. (2023) employed self-reported data on lifestyle modification, fear, stress, and help-seeking behaviors, whereas Every-Palmer et al. (2024) utilized structured survey items without standardized measurement instruments.

Furthermore, the aforementioned lack of validation was also applicable to the categories “exposure” and “condition,” which were marked as “unclear” for all studies. This is due to the absence of standardized scales for assessing TFGBV experiences at the time of this review, which is a consequence of the nascent stage of this field. For example, although Adams (2018) and Carlson and Witt (2020) employed a focus on self-reported data to capture exposure to online harassment, the lack of validated scales for measuring both the frequency and severity of threats faced by female journalists constrained the studies’ ability to ensure the validity and reliability of these measures. Furthermore, the heterogeneity of measurements used across studies represents a limitation. In the absence of a universally accepted benchmark for evaluating TFGBV in this context, the metrics employed are predominantly disparate and grounded in subjective experiences. Consequently, the findings of these studies should be interpreted with caution, given their exploratory nature and inherent biases in self-reported data.

Supplemental Table S4 presents the critical appraisal of the qualitative studies, employing the JBI tool to evaluate five key quality indicators. The majority of studies, including those by Baloch et al. (2025), Nelson (2022), and others, demonstrated satisfactory performance in the areas of recruitment, data collection, and ethical considerations. However, a significant limitation across the majority of the studies was the lack of explicit discussion regarding the relationship between the researcher and participants. Baloch et al. (2025) was the only study to achieve a high score in all categories, addressing the researcher’s positionality and potential influence. Additionally, only 13 of the 27 qualitative studies stated that they had obtained ethical approval from an institutional review board or similar entity, indicating a lack of consistent adherence to ethical standards across the studies. While many studies demonstrated effective management of data collection and ethical considerations, the absence of reflection on the researcher’s influence remained a persistent issue. The risk of bias appraisal for the 11 mixed-method studies can be found in Supplemental Table S4. The findings are consistent with the patterns identified in the appraisal of both the quantitative and qualitative studies. It is notable that only a few studies accounted for confounding factors, namely those conducted by Bauschke and Jäckle (2023) and Erikson et al. (2021). Furthermore, positionality considerations were not addressed in any of the studies. To ensure inclusivity and contextual relevance, Posetti and Shabbir (2022) translated their survey into five languages: Arabic, English, French, Portuguese, and Spanish.

## Discussion and Conclusion

The findings of this systematic review offer critical insights into how politically active women navigate the challenges posed by TFGBV. By synthesizing in-depth data from 48 studies, this review not only confirms the profound emotional, psychological, and professional toll of online harassment but also highlights the diversity of responses to these attacks. Politically active women employ a range of coping mechanisms depending on their individual circumstances, resources, and the severity of the harassment they face. The majority of the studies included in this review are concentrated in Western and anglophone contexts, particularly the United States and the United Kingdom, while research from non-Western regions remains scarce. However, several cross-country studies offer country comparisons and selected country studies offer valuable insights into unique contexts. For example, Kim and Shin (2022) explore the growing mistrust of journalists in South Korea, which impacts their experience with online harassment. Furthermore, Tandoc et al. (2023) highlight how state actors in the Philippines play a role in initiating harassment, contributing to climates of misinformation and oppression against female journalists. These context-specific findings underscore the different environments politically active women find themselves in when dealing with online harassment.

### Psychological Consequences

The systematic review demonstrated that TFGBV had a significant emotional and psychological impact on female politically active women, encompassing a spectrum of reactions from initial confusion and frustration to behaviors indicative of suicidality in extreme cases. Therefore, it is imperative to consider the cumulative effect of online harassment. The considerable quantity of harassment, coupled with the iterative strategies required to prevent or mitigate attacks, results in substantial psychological exhaustion. As Tandoc et al. (2023) suggests, while emotional coping may offer temporary alleviation, repeated attacks can have detrimental long-term effects on women’s well-being, underscoring the necessity for future research into the prolonged impact of such harassment. Furthermore, the anticipation of future attacks engenders a pervasive state of apprehension, markedly disrupting women’s daily lives and contributing to long-lasting psychological harm.

Finally, the emotional and psychological implications of these consequences extend beyond the immediate context. The impact of online harassment is not limited to the current generation of women; many politically active women also experience a double burden, concerned about the potential consequences of harassment on younger or aspiring women. Seven of the studies included in this review documented how women feel a responsibility to challenge sexism for future generations. This concern illustrates how the experienced silencing effect extends into worrying about a chilling effect on women who have not yet been attacked, discouraging their full participation.

### Political Consequences

The impact of TFGBV on women’s professional lives is both immediate and long-lasting. The review revealed that harassment frequently results in substantial alterations in the manner in which women approach their work. Many are compelled to modify the tone, complexity, or content of their communications in order to circumvent further attacks. This additional labor not only detracts from their productivity but also highlights the disparate expectations placed upon women in public and political roles, where the legitimacy of their voices is frequently unfairly subjected to unwarranted scrutiny in response to harassment.

The findings of this systematic review suggest that politically active women are often forced to balance the risks of engagement with the emotional toll of harassment, leading many to alter their public personas or communication strategies, which can perpetuate the status quo, as fewer women participate fully in digital conversations. The professional consequences are equally stark, with many women experiencing reputational damage, productivity losses, and even considering or pursuing career changes as a direct result of online. This silencing effect on political, activist, and journalistic discourse has serious implications for democracy, as it limits the range of voices contributing to public debates (E. Jane, 2018; Ferrier, 2018; Koch et al., 2024).

### Coping Mechanisms

Findings from this review illustrate that women’s responses to TFGBV are far from monolithic, with different strategies reflecting the complexity of coping with harassment in digital spaces. Many women adopt strategies, such as enhancing digital security, reporting incidents to platforms or authorities, or taking legal action, which aligns with preventive labor as identified by Sobieraj (2021). This labor imposes an additional burden on women, further complicating their professional routines. While these measures may offer temporary relief or a sense of control, they are resource-intensive, time-consuming, and emotionally draining, illustrating how TFGBV generates unpaid, invisible labor that disproportionately affects women.

Furthermore, purposeful ignoring emerged as a common tactic, with women deliberately avoiding reading comments or engaging with hostile content to preserve their mental well-being. However, this response, while psychologically protective, also underscores the silencing effect of TFGBV. By refraining from engaging fully in digital spaces or by suffering irreparable reputational damage, women are “forced out” (instead of “opting out”), reducing the diversity of voices and ideas in political and professional spheres.

The coping strategies available to women are shaped by their intersecting identities. Ethnicity, age, and sexual orientation play critical roles in determining not only how women respond to harassment but also the support systems and resources they can draw on to protect themselves.

With this in mind, the review also identified a dual narrative in how women perceive and respond to TFGBV. On the one hand, many women view harassment as a cost of their profession, accepting it as an inevitable consequence of being a woman in the public eye. On the other hand, fewer women actively resist this narrative, engaging in feminist “digilantism” or other forms of public confrontation as a way to reclaim their agency and resist the silencing effects of online abuse (Chen et al., 2018; E. Jane, 2018; Kantola & Harju, 2023). However, as Jane (2018) points out, this shifts the responsibility for managing the abuse onto the targets themselves, rather than addressing the root causes of platform structures or holding perpetrators accountable.

One limitation of this systematic review is the exclusive use of English-language sources, which may have led to the exclusion of region-specific studies and important insights from non-English-speaking contexts. This limitation is particularly relevant given growing evidence that TFGBV may manifest differently in autocratic or conflict-affected settings. In such contexts, online harassment is intertwined with broader state-sponsored strategies of repression, surveillance, and disinformation (Posetti & Shabbir, 2022; Tandoc et al., 2023). These environments can shape not only the frequency and severity of TFGBV but also the range of available coping mechanisms, as women may lack institutional protections or face heightened risks when speaking out. However, academic research in these settings is frequently constrained by safety concerns and limited access to reliable data (Article 19, 2021). As a result, important insights into the lived experiences of politically active women in these regions remain underrepresented in the current evidence base. Additionally, only 25% of the included studies accounted for intersectionality characteristics. Future research should explore how regional or subnational variation within countries—such as differences in political, cultural, or religious norms—may shape women’s vulnerability to and experiences of TFGBV.

Another limitation is the variation in sampling and data collection methods, which makes it difficult to compare and contrast study findings.

A further limitation concerns the distribution of technological platforms examined across the included studies. While the search strategy captured a wide range of digital tools and environments, the majority of studies focused on harassment facilitated through social media and messaging applications. As a result, forms of TFGBV occurring on less visible or less studied platforms—such as encrypted messaging services, gaming platforms, or stalking devices—are underrepresented. Future studies should expand attention to underexplored digital environments in order to better understand how different technological infrastructures shape experiences of gender-based violence.

Finally, a limitation concerns the over-representation of journalists in the sample, as most studies report findings primarily from this population group. This focus on journalists impedes generalizability, as it remains unclear whether the observed reactions and coping mechanisms are representative of other professional groups, such as politicians, who may experience different levels of institutional support or public scrutiny.

### Implications and Conclusion

This review highlights several critical implications for platforms, policymakers, and public institutions committed to gender equity in digital and political spaces. Addressing TFGBV requires more than individual coping strategies—it demands structural changes. The current system places the burden of managing abuse on women themselves, reinforcing gendered labor inequities and perpetuating a toxic digital environment. Long-term solutions must include improved platform governance, institutional accountability, and legal protections that specifically address the needs of women in public-facing roles- particularly those from marginalized backgrounds. In particular, this review has shown that addressing forms of TFGBV that manifest as economic or physical violence presents unique challenges. These harms are often indirect, cumulative, and difficult to isolate—such as reputational damage that quietly affects career advancement, or online threats that result in changes to personal security precautions. Because these consequences are less visible and often not recognized in institutional policies, they frequently fall through the cracks of support systems and legal frameworks. More holistic and intersectional responses are needed to account for the full scope of these impacts.

Additionally, this review underscores the geographic imbalance in current research. This review highlights the scarcity of research from non-Western contexts, which highlights the pressing need for studies that examine the experiences of politically active women in the Global South. Given that online spaces are critical to political participation and economic advancement, ensuring women’s safe and equitable access to these spaces is paramount for global development. Future research should aim to center these perspectives and examine the intersectional vulnerabilities shaped by race, ethnicity, language, and access to institutional support. In addition, further research could explore less visible trauma responses—such as “freeze” and “fawn”—which are well-established in trauma theory but have not yet been systematically investigated in the context of TFGBV. Including these responses may provide a more comprehensive understanding of how online abuse is experienced by women who do not- or cannot-respond through confrontation or withdrawal.

In sum, TFGBV not only harms individual women but also weakens societal structures by eroding women’s professional vitality, limiting their political engagement, and constraining their economic potential. As digital spaces continue to evolve, it is crucial to ensure that they are inclusive, safe, and supportive of diverse voices—especially those of women, who are essential drivers of democratic and development processes worldwide.

## Supplemental Material

sj-docx-1-tva-10.1177_15248380251343185 – Supplemental material for Technology-Facilitated Gender-Based Violence Against Politically Active Women: A Systematic Review of Psychological and Political Consequences and Women’s Coping Behaviors Supplemental material, sj-docx-1-tva-10.1177_15248380251343185 for Technology-Facilitated Gender-Based Violence Against Politically Active Women: A Systematic Review of Psychological and Political Consequences and Women’s Coping Behaviors by Luise Koch, Maria Paula Russo Riva and Janina Isabel Steinert in Trauma, Violence, & Abuse
