# Supplementary material for: Technology-Facilitated Gender-Based Violence Against Politically Active Women: A Systematic Review of Psychological and Political Consequences and Women’s Coping Behaviors
Source: Trauma Violence Abuse. 2025 Jun 27;27(4):1231–50. doi: 10.1177/15248380251343185 (PMC13578654; doi:10.1177/15248380251343185)
Supplement: Supplementary file 1 [file sj-docx-1-tva-10.1177_15248380251343185.docx]

**Supporting Materials**

Technology-Facilitated Gender-Based Violence against Politically Active Women: A Systematic Review of Psychological and Political Consequences and Women's Coping Behaviors

This supplementary file includes the following:

[Title & abstract screening inclusion guide	2](#_Toc182128701)

[Paper inclusion guide	5](#_Toc182128702)

[Figure S1. Detailed title and abstract screening	11](#_Toc182128703)

[Table S1. Cohen’s Kappa Calculation	11](#_Toc182128704)

[Table S2. Detailed subcategories of outcome variables	11](#_Toc182128705)

[Table S3. Critical appraisal for included cross-sectional studies	15](#_Toc182128706)

[Table S4. Critical appraisal for included qualitative studies	16](#_Toc182128707)

[Table S5. Critical appraisal for included mixed-methods studies	18](#_Toc182128708)

Appendix:

This supplementary file provides additional details on the systematic review process and outcomes related to how politically active women react to technology-facilitated gender-based violence. It includes the title and abstract screening inclusion guide, the paper inclusion guide, and Figure S1, which illustrates the detailed title and abstract screening process. Table S1 presents the Cohen’s Kappa calculation used to assess inter-rater reliability, while Table S2 provides detailed subcategories of the outcome variables. Table S3- S4 presents the risk of bias appraisal for the included studies depending on the methods used.

# Title & abstract screening inclusion guide

**October -’23**

**Overall:**

- Please screen the titles and abstracts of the papers and decide whether they should be considered for the full-text screening process.
- In order to decide on the inclusion of the studies for the paper screening, please conduct the screening with the PEO framework in mind. I.e. Include at least one criterion of each group of the PEO framework:
  - **P**opulation: e.g. women, activists, journalists, politicians, feminists;
  - **E**xposure: e.g. TFGBV, ICT, online hate, cybercrime, ICT;
  - **O**utcome: e.g. mental health, reaction.

See the complete list of **Inclusion Criteria** below

- Rayyan allows you to either “include”, “exclude” or “indecisive”. In case you chose an “undecisive” criterion, please add a note through the Rayyan note function and “flag” for discussion or review.
- You might want to use the highlight text function to highlight specific pieces of information being extracted (for easy review and checking in the second stage of extraction) but this is not compulsory.
- The four of us will all screen the same 100 abstracts & titles independently and meet afterward to discuss discrepancies and align the guidelines.
- Consequently, Becky and I will double-screen 10% of the sample (i.e. 1.594 titles & abstracts) to calculate an inter-coder agreement score. The remaining 90% of the sample will be distributed amongst us; i.e. each one of us will screen 3.586 titles & abstracts.

**Inclusion Criteria**

|  | **Inclusion Criteria** | **Exclusion Criteria** |
| --- | --- | --- |
| **Population** | Politically active (transgender) women aged above 18 such as   - candidates for office, - politicians, - government officials, - journalists, - activists, - human rights defenders, - social leaders and - female scientists with public appearances. | Women who are targets of online violence but who are not politically active e.g. adolescents; and politically active men will be excluded.  Studies including mixed-gender samples will only be included if results are disaggregated for male and female participants |
| **Exposure** | Studies that include technology-facilitated gender-based violence (TFGBV) as an exposure. TFGBV is thereby broadly defined and includes aspects of   - ***psychological violence***: e.g., threats of /intimidation with (sexual/physical) violence intended to cause fear; mobbing; trolling; spamming such as continuously contacting, annoying, threatening, and/ or scaring someone; sending unsolicited (sexual) images or content; flaming e.g., posting deliberately inflammatory of off-topic material to humiliate and/or provoke a response or emotional reaction; distribution of gendered disinformation, - ***sexual violence:*** e.g., non-consensual distribution of sexual images (e.g., revenge pornography); image-based abuse (e.g., generation and distribution of deep fake sexual images or videos); cyber flashing (e.g., sending someone sexually explicit imagery of themselves (or others) without consent); defamation based on sexuality and sexual behaviour; creating and disseminating content/false information which attacks or undermines people based on their gender; rape threats, - ***economic violence:*** e.g., hacking a bank account or other resources of targets to gain unauthorized access to systems or information; forcing the target to further invest in their (families/friends) security, - ***physical violence:*** e.g., surveillance/tracking/cyberstalking or use of technology to track/stalk/monitor the activities/physical location of a person to commit an assault; hate speech inciting physical violence or undermining participation in the public sphere; doxing in the sense of publicizing a person’s home address with the intent that others may cause them harm; recruitment: the use of technology to lure people into potentially violent situations (e.g., traffickers using chat rooms) and - ***political/symbolical/reputational violence:*** e.g., impersonating (e.g., using technology to assume the identity of someone else, e.g., creating a fake profile for social networking sites) a person to damage their reputation or credibility; doxing (disclosing personal information to undermine a person’s credibility and/ or reputation,   which are committed in part or fully, by the use of Internet and Communication Tools (ICTs) or other digital tools, such as mobile phones, GPS/location-based technologies, social media platforms (i.e., Twitter, Instagram, Facebook) and email.  There will be no further constraints on the type, duration or content of the exposure. | Studies that do not include any of the aspects of TFGBV mentioned in the inclusion part. |
| **Outcome** | The first outcome is the reactions from targets of online abuse. We will adopt a broad definition of possible responses to TFGBV and include outcome measures that fit into one of the following two categories: psychological reactions and political reactions.  ***Psychological reactions***:   - Feeling embarrassed/ashamed - Anxiety - Psychological distress - Depression - Low self-esteem/self-depreciation/loss of self-confidence - Self-harm - Post-traumatic stress disorder - Suicidality - Concerns about one’s own/family’s/friends’ safety   ***Political reactions***:   - Changing the frequency of online platform use - Changing the type/content/tone of political communications (offline and online) - Changing the frequency of offline appearances - Productivity losses (e.g., missing work, taking sick leave, etc.) - Withdrawal from politics (e.g., quitting one’s job)   The second outcome is the target’s coping strategies for online abuse.  ***Coping strategies***:   - Increasing security measures online - Increasing security measures offline - Socialising - Other self-care | Studies that do not include any of the outcomes mentioned in the inclusion part. |

*Note:* We will include any qualitative report and quantitative estimate of relevant responses.

# Paper inclusion guide

- **May ‘24**

**Overall:**

·	Use UK (rather than US spelling)

·	If you think the study does not qualify – fill in at least until the study title (A – F) and change to red text for further review

·	When papers (studies) present cross-country results, treat each country as its own study, unless results are only presented (analyzed) pooled, then they are the same study, and we can discuss how to code the study level information

·	Use the highlight text function to highlight specific pieces of information being extracted (for easy review and checking in the second stage of extraction)

·	Any other items to “flag” for discussion or review – put in red text

**Study level guidance**

| **Col** | **Item** | **Notes** |
| --- | --- | --- |
| **A** | Data Extractor | Use drop-down  LK - review all studies  MP - double screen 10% and check extractions for remaining 90% |
| **B** | Date of Extraction | Please insert the date you conducted the data extraction on |
| **C** | Study ID | First digit(s) of the study file name – e.g. 002, 003, etc. |
| **D** | Authors | Should be in typical citation format, e.g.  · Peterman (if only 1 author)  · Peterman & Steinert (if 2 authors)  · Baird et al. (if 3 or more authors) |
| **E** | Year | Publication date e.g., 2021, 2022 etc. |
| **F** | Study Title | Enter exactly as in on the publication, no quotes or other text—do not capitalise words other than the title's first word |
| **G** | Publication Type | Use drop down |
| **H** | Country of Study | Full name, using English spelling  If countries are not specified, state “various”. These studies will be included in the final visualization map with dashed lines only if the countries are specifically mentioned. |
| **I** | Region | Use WB classifications:  <https://datahelpdesk.worldbank.org/knowledgebase/articles/906519-world-bank-country-and-lending-groups> |
| **J** | Sample Size | Indicate the number of participants in the study |
| **K** | Study Participants: Political Activity | Use drop-down |
| **L** | Study Participants: Age | Mean years of the analysis sample up to one decimal point (e.g., 27.7). “No information” if no age information was provided.  Note: Studies qualify for inclusion if the sample is either exclusively women or 50% or more women and if the data is, in such cases, gender disaggregated. |
| **M** | Study Participants: Intersectionality Characteristic | Stated intersectionality as a focus of the study sample – only include if the study explicitly mentions these dimensions (do not infer based on broader context):    · **Gender Identity**: Select if the study indicates gender-identity of study participants as a reason for the received attacks online.  · **Sexual Orientation**: Select if the study indicates the sexual orientation of study participants as a reason for the received attacks online.  · **Age**: Select if the study indicates a certain age (too young/too old) of study participants as a reason for the received attacks online.  · **Disability**: Select if the study indicates certain disability characteristics of study participants as a reason for the received attacks online.  · **Feminism**: Select if the study indicates feminist views of study participants as a reason for the received attacks online  · **Ethnicity**: Select if the study indicates a certain ethnic minority (e.g. indigenous or other caste-based targeting) of study participants as a reason for the received attacks online.  – select “not considered” if the study does not focus on participants selected based on any intersectionality characteristic.    If the study gives more than one targeting focus, select all. |
| **N** | Method of Study | Use drop-down |
| **O** | Data Used | Indicate which kind of data was used for a basis of analysis (e.g., survey responses, qualitative content analysis of conducted interviews) |
| **P** | Outcome Variable (1): psychological outcomes/measures | Stated **psychological consequences:**   - Initial Confusion/Surprise - Unpreparedness/Helplessness/Impotence/Vulnerability/Overwhelm - Isolation/Loneliness - Frustration/Anger/Annoyance/Upset/Disgust/Offense/Discouragement - Shame/Embarrassment/Devaluation/Self-Punishment for Inaction - Psychological Distress/Anxiety/Fear/Shock/Discomfort/Intimidation (e.g., Panic Attacks, Difficulty Sleeping, Heightened Anxiety.) - Low Self-Esteem/Self-Deprecation/Loss of Confidence/Embarrassment/Shame - Unhealthy Habits (e.g., Shopping Addiction/Gaming Addiction) - Depression - Post-Traumatic Stress Disorder/Re-traumatization (e.g., Experiencing Trauma from Harassment, Panic Attacks, Triggering of Past Abuse, Physical Symptoms of Trauma) - Suicidality/Self-harm - Personal Safety Concerns - Safety Concerns about Family/Friends/Staff - Concerns for Future Generations (e.g., Worrying About Impact of Harassment on Young or Aspiring Women, Feeling Responsible to Challenge Sexism for Future Generations) - Fear of Professional Repercussions (e.g., Concerns About Reporting Harassment Leading to Dismissal or Disregard, Fear of Being Labeled a Troublemaker, Worrying About Harm to Career Advancement or Professional Relationships) - Impact on Close Relationships (e.g., Family Members Becoming Worried or Scared, Physical Attacks on Family Members, Threats Extending to Family, Negative Effects on Relationships) - Motivation/Empowerment/Defiance (e.g. Feeling Driven to Take Action, Standing up to Harassers.) - Emotional Numbness (e.g., Disengagement, Feeling Neutral, Lack of Emotional Response) - Indecision/Lack of Direction (e.g., Uncertainty on how to Proceed, Confusion About Appropriate Response)   For qualitative studies extract direct quotations from interviews/FGDs. |
| **Q** | Outcome Variable (2): political outcomes/measures | Stated **political/professional consequences**:   - Decrease in Online Platform Use - Increase in Online Platform Use - Changes in Political Communication (Content, Tone, Complexity, Depth, Perfectionism) - Decrease in Offline Appearances - Platform Migration - Account/Function Closure (e.g., Messenger) - Productivity Losses (e.g., Missed Work, Sick Leave, Cognitive Load, Reputational Harm, Disruption in Information Gathering) - Thoughts of Withdrawal from Job - Absence of Withdrawal Thoughts from Job - Job Withdrawal - Firing/Suspension/Firing/Reprimand/Told to Toughen Up - Keeping Political Communication/Working Routine   For qualitative studies, extract direct quotations from interviews/FGDs. |
| **R** | Outcome Variable (3): coping strategies/behaviors | Stated **coping strategies/behaviors**   - Increase in Online Security (e.g., Disabling Comments/Notifications, Hiding Personal Information, Changing Passwords, Using Anonymous Identities, Modifying Profile Pictures/Names, Using Spam Filters or Blocklists) - Increase in Offline Security (e.g., Changing Routes, Walking with Companions, Avoiding Traveling Alone in the Dark, Avoiding Ground-Floor Residences, Hypervigilance During Public Events, Relocation, Involvement of Police) - Blocking/Muting/Ignoring/Non-Response to Abuse - Abstention from Blocking - Abstention from Comment Reading - Original Post Deletion - Abuse Deletion (Attempted) - Reporting Attempts - Reporting - Non-Reporting - Inability to Report - Reading Comments Fully/Attempts to Understand Harassers (e.g., Researching Harassers, Perspective-Taking, Rationalizing Harassers' Behavior) - Private Comment Replies - Online Confrontation of Perpetrators (e.g., Public Replies, Exposing Harassers, Engaging in Discussions, Threatening Legal Action, Replying Harshly) - Offline Confrontation of Perpetrators (e.g., Tracking and Contacting Perpetrators Directly, Visiting Their Workplaces, Involving Family Members or Employers) - Online Discussion for Support (e.g., Publicly Sharing Abuse to Raise Awareness, Blogging or Posting About Harassment, Seeking Support from Followers) - Collaborative Support Networks and Monitoring (e.g., Asking Colleagues to Block Offenders or Reply to Harassment, Setting Up Social Media Teams, Collaboration with Advocates, Community Groups, and Specialists) - Social Support and Building Allies (e.g., Discussing Harassment with Friends, Family, or Colleagues, Forming Support Networks, Sharing Incidents for Emotional Support, Building (cross-national) Allies, Peer Assistance in Moderating or Handling Comments) - Psychological Support Seeking - Professional Support Seeking (e.g., Boss, Supervisor, Colleagues) (Seeking Assistance with Harassment Management, Requesting Emotional or Practical Support) - Personal Self-Care Practices (e.g., Dealing with Harassment Individually, Seeking Spiritual or Mental Guidance) - Normalization and Rationalization of Harassment (e.g., Developing a Thick Skin, Shrugging it off, Accepting Harassment as Part of the Job, Downplaying Abuse, Building Emotional Resilience) - Humor as a Coping Mechanism (e.g., Using Humor to Deflate Negative Comments, Creating "Wall of Shame," Playfully Responding to Harassment, Sharing and Joking About Abusive Messages with Colleagues, Making Light of Vile Comments) - Refusing to Normalize Harassment (e.g., Expressing Outrage at Acceptance of Abuse, Challenging the Notion that Harassment "Comes with the Job")   For qualitative studies, extract direct quotations from interviews/FGDs in *italics*. Everything else in “normal”. |
| **S** | Exposure Variable | Indicate which kind of online hate the study looked at. E.g. harassment and sexual harassment, threats of physical and sexual violence, flaming and trolling, stalking, electronic sabotage, impersonation and defamation or whatever the study looks at. |
| **T** | Summary of Results | In light of our PEO |
| **U** | Context | Indicate whether the study had the aim to analyse a certain context (i.e. election), if not indicate “no specific context”. |
| **V** | Abstract | Cut and paste the abstract |
| **W** | Additional Information |  |
| **X** | Comments/Questions | On coding questions or other responses |
| **Y** | Checked by | Initials of the person who checked the entry |

# Figure S1. Detailed title and abstract screening

*Note:* A1-A3 indicate the authors of the paper which are anonymized

# Table S1. Cohen’s Kappa Calculation

|  |  | **A3** | |  |
| --- | --- | --- | --- | --- |
|  |  | Include | Exclude | Total |
| **A1** | Include | 2 | 1 | 3 |
|  | Exclude | 0 | 1591 | 1591 |
|  | Total | 2 | 1592 | **1594** |

*Note:* Where p0=0.99937, pe=0.99686 and Kappa=0.799698 indicating a substantial agreement

# Table S2. Detailed subcategories of outcome variables

| **Outcome Variable 1: Psychological consequences** | Initial Confusion/Surprise |
| --- | --- |
|  | Unpreparedness/Helplessness/Impotence/Vulnerability/Overwhelm |
|  | Isolation/Loneliness |
|  | Frustration/Anger/Annoyance/Upset/Disgust/Offense/Discouragement |
|  | Shame/Embarrassment/Devaluation/Self-Punishment for Inaction |
|  | Psychological Distress/Anxiety/Fear/Shock/Discomfort/Intimidation (e.g., Panic Attacks, Difficulty Sleeping, Heightened Anxiety.) |
|  | Low Self-Esteem/Self-Deprecation/Loss of Confidence/Embarrassment/Shame |
|  | Unhealthy Habits (e.g., Shopping Addiction/Gaming Addiction) |
|  | Depression |
|  | Post-Traumatic Stress Disorder/Re-traumatization (e.g., Experiencing Trauma from Harassment, Panic Attacks, Triggering of Past Abuse, Physical Symptoms of Trauma) |
|  | Suicidality/Self-harm |
|  | Personal Safety Concerns |
|  | Safety Concerns about Family/Friends/Staff |
|  | Concerns for Future Generations (e.g., Worrying About Impact of Harassment on Young or Aspiring Women, Feeling Responsible to Challenge Sexism for Future Generations) |
|  | Fear of Professional Repercussions (e.g., Concerns About Reporting Harassment Leading to Dismissal or Disregard, Fear of Being Labeled a Troublemaker, Worrying About Harm to Career Advancement or Professional Relationships) |
|  | Impact on Close Relationships (e.g., Family Members Becoming Worried or Scared, Physical Attacks on Family Members, Threats Extending to Family, Negative Effects on Relationships) |
|  | Motivation/Empowerment/Defiance (e.g. Feeling Driven to Take Action, Standing up to Harassers) |
|  | Emotional Numbness (e.g., Disengagement, Feeling Neutral, Lack of Emotional Response) |
|  | Indecision/Lack of Direction (e.g., Uncertainty on how to Proceed, Confusion About Appropriate Response) |
| **Outcome Variable 2: Political Outcomes** | Decrease in Online Platform Use |
|  | Increase in Online Platform Use |
|  | Changes in Political Communication (Content, Tone, Complexity, Depth, Perfectionism) |
|  | Decrease in Offline Appearances |
|  | Platform Migration |
|  | Account/Function Closure (e.g., Messenger) |
|  | Productivity Losses (e.g., Missed Work, Sick Leave, Cognitive Load, Reputational Harm, Disruption in Information Gathering) |
|  | Thoughts of Withdrawal from Job |
|  | Absence of Withdrawal Thoughts from Job |
|  | Job Withdrawal |
|  | Firing/Suspension/Firing/Reprimand/Told to Toughen Up |
|  | Keeping Political Communication/Working Routine |
| **Outcome Variable 3: Coping Mechanisms** | Increase in Online Security (e.g., Disabling Comments/Notifications, Hiding Personal Information, Changing Passwords, Using Anonymous Identities, Modifying Profile Pictures/Names, Using Spam Filters or Blocklists) |
|  | Increase in Offline Security (e.g., Changing Routes, Walking with Companions, Avoiding Traveling Alone in the Dark, Avoiding Ground-Floor Residences, Hypervigilance During Public Events, Relocation, Involvement of Police) |
|  | Blocking/Muting/Ignoring/Non-Response to Abuse |
|  | Abstention from Blocking |
|  | Abstention from Comment Reading |
|  | Original Post Deletion |
|  | Abuse Deletion (Attempted) |
|  | Reporting Attempts |
|  | Reporting |
|  | Non-Reporting |
|  | Inability to Report |
|  | Reading Comments Fully/Attempts to Understand Harassers (e.g., Researching Harassers, Perspective-Taking, Rationalizing Harassers' Behavior) |
|  | Private Comment Replies |
|  | Online Confrontation of Perpetrators (e.g., Public Replies, Exposing Harassers, Engaging in Discussions, Threatening Legal Action, Replying Harshly) |
|  | Offline Confrontation of Perpetrators (e.g., Tracking and Contacting Perpetrators Directly, Visiting Their Workplaces, Involving Family Members or Employers) |
|  | Online Discussion for Support (e.g., Publicly Sharing Abuse to Raise Awareness, Blogging or Posting About Harassment, Seeking Support from Followers) |
|  | Collaborative Support Networks and Monitoring (e.g., Asking Colleagues to Block Offenders or Reply to Harassment, Setting Up Social Media Teams, Collaboration with Advocates, Community Groups, and Specialists) |
|  | Social Support and Building Allies (e.g., Discussing Harassment with Friends, Family, or Colleagues, Forming Support Networks, Sharing Incidents for Emotional Support, Building (cross-national) Allies, Peer Assistance in Moderating or Handling Comments) |
|  | Psychological Support Seeking |
|  | Professional Support Seeking (e.g., Boss, Supervisor, Colleagues) (Seeking Assistance with Harassment Management, Requesting Emotional or Practical Support) |
|  | Personal Self-Care Practices (e.g., Dealing with Harassment Individually, Seeking Spiritual or Mental Guidance) |
|  | Normalization and Rationalization of Harassment (e.g., Developing a Thick Skin, Shrugging it off, Accepting Harassment as Part of the Job, Downplaying Abuse, Building Emotional Resilience) |
|  | Humor as a Coping Mechanism (e.g., Using Humor to Deflate Negative Comments, Creating "Wall of Shame," Playfully Responding to Harassment, Sharing and Joking About Abusive Messages with Colleagues, Making Light of Vile Comments) |
|  | Refusing to Normalize Harassment (e.g., Expressing Outrage at Acceptance of Abuse, Challenging the Notion that Harassment "Comes with the Job") |

# Table S3. Critical appraisal for included cross-sectional studies

| **Study** | **Sampling** | **Participants** | **Confounding** | **Measure** | **Analysis** |
| --- | --- | --- | --- | --- | --- |
| Carlson & Witt (2020) | x | x |  |  | x |
| Phillips *et al* (2023) | x | x |  |  |  |
| Lee & Park (2023) | x | x | x | x | x |
| Stahel & Schoen (2020) | x | x | x | x | x |
| Adams (2017) | x |  |  |  | x |
| Akhtar & Morrison (2019) | x | x |  |  | x |
| Lewis (2020) | x | x | x | x | x |
| Celuch *et al* (2022) | x | x | x | x | x |
| Celuch *et al* (2023) | x | x |  | x | x |
| Every-Palmer *et al* (2024) | x | x |  |  | x |

*Note:* The coding is based on the Joanna Briggs Institute tool for the appraisal of cross-sectional quantitative studies. The two categories on “Were confounding factors identified?” and “Were strategies to deal with confounding factors stated?” were collapsed into one column and are marked with “x” if reported in the respective study. The category on exposure (“Was the exposure measured in a valid and reliable way?”) and the category on the condition (“Were objective, standard criteria used for measurement of the condition?”) were classified as “unclear” for all the studies as no existing scale to assess TFGBV experiences exists at the time of writing this article.

# Table S4. Critical appraisal for included qualitative studies

| **Study** | **Recruitment** | **Data collection Method** | **Relationship between participants and researchers** | **Ethics** | **Analysis** |
| --- | --- | --- | --- | --- | --- |
| Nelson (2020) | x | x |  | x | x |
| Zviyita & Mare (2023) | x | x |  |  | x |
| Sampaio-Dias *et al* (2023) | x | x |  |  | x |
| Chen *et al* (2018) | x | x |  | x | x |
| Walulya & Selnes (2023) |  | x |  |  | x |
| Wagner (2022) | x | x |  | x | x |
| Pain & Chen (2019) | x | x |  |  | x |
| Koirala (2020) | x | x |  |  | x |
| Goyal *et al* (2022) | x | x |  | x | x |
| Article 19 (2021) | x | x |  |  | x |
| Sarikakis *et al* (2021) | x | x |  |  | x |
| Eckert (2018) | x | x |  | x | x |
| García & Sequera (2021) | x | x |  |  | x |
| Jane (2018) | x | x |  | x | x |
| Kim & Shin (2022) | x | x |  |  | x |
| Tandoc *et al* (2021) | x | x |  | x | x |
| Kundu *et al* (2021) | x | x |  |  | x |
| Miller & Lewis (2020) | x | x |  |  | x |
| Uwalaka & Amadi (2023) | x | x |  |  | x |
| Holton *et al* (2021) | x | x |  | x | x |
| Deavours *et al* (2022) | x | x |  | x | x |
| Sobieraj (2021) | x | x |  | x | x |
| Harlow *et al* (2023) | x | x |  | x | x |
| Barão da Silva *et al* (2022) | x | x |  | x | x |
| Kantola & Harju (2021) | x | x |  |  | x |
| Smith (2019) | x | x |  |  | x |
| Baloch *et al* (2024) | x | x | x | x | x |

*Note:* The coding is based on the Joanna Briggs Institute tool for the appraisal of qualitative studies. We have highlighted five out of ten items of the tool and marked them with “x” if reported in the respective study. The two categories “Is there a statement locating the researcher culturally or theoretically?” and “Is the influence of the researcher on the research, and vice-versa, addressed?” were collapsed into one column and are marked with “x” if reported in the respective study.

# Table S5. Critical appraisal for included mixed-methods studies

|  | *Categories from the JBI tool for the appraisal of cross-sectional quantitative studies* | | | | | *Categories from the JBI tool for the appraisal of qualitative studies* | | | | |
| --- | --- | --- | --- | --- | --- | --- | --- | --- | --- | --- |
| **Study** | **Sampling** | **Participants** | **Confounding** | **Measure** | **Analysis** | **Recruitment** | **Data collection method** | **Relationship between participants and researchers** | **Ethics** | **Analysis** |
| Kempton & Connolly-Ahern (2022) | x | x |  |  |  | x | x |  | x | x |
| Erikson *et al* (2021) | x | x | x |  | x | x | x |  |  | x |
| Binns (2017) | x | x |  |  | x | x | x |  |  | x |
| Bauschke & Jäckle (2023) | x | x |  |  | x | x | x |  | x | x |
| Sequera & Acuña (2023) | x | x |  |  |  | x | x |  | x | x |
| Lewis *et al* (2016) | x | x |  |  | x | x | x |  | x | x |
| Lekić-Subašić (2023) | x | x |  |  | x | x | x |  |  | x |
| Jovanovska (2022) | x | x |  |  | x | x | x |  |  | x |
| Ferrier (2018) |  | x |  |  | x | x | x |  | x | x |
| Carson *et al* (2023) | x | x | x |  | x | x | x |  | x | x |
| Posetti & Shabbir (2022) | x | x | x |  | x | x | x |  | x | x |
